# Noninvasive Deep Brain Stimulation via Temporally Interfering Electric Fields

**DOI:** 10.1016/j.cell.2017.05.024

**Published:** 2017-06-01

**Authors:** Nir Grossman, David Bono, Nina Dedic, Suhasa B. Kodandaramaiah, Andrii Rudenko, Ho-Jun Suk, Antonino M. Cassara, Esra Neufeld, Niels Kuster, Li-Huei Tsai, Alvaro Pascual-Leone, Edward S. Boyden

**Affiliations:** 1 Media Lab, MIT, Cambridge, MA 02139, USA; 2 McGovern Institute for Brain Research, MIT, Cambridge, MA 02139, USA; 3 Berenson-Allen Center for Noninvasive Brain Stimulation, Department of Neurology, Beth Israel Deaconess Medical Center, Harvard Medical School, Boston, MA 02215, USA; 4 Centre for Bio-Inspired Technology, Department of Electrical and Electronic Engineering, Imperial College London, SW7 0AZ London, UK; 5 Department of Materials Science and Engineering, MIT, Cambridge, MA 02139, USA; 6 Department of Mechanical Engineering, University of Minnesota, Twin Cities, Minneapolis, MN 55455, USA; 7 Picower Institute for Learning and Memory, MIT, Cambridge, MA 02139, USA; 8 Department of Biology, City College of the City University of York, New York, NY 10031, USA; 9 IT’IS Foundation for Research on Information Technologies in Society, 8004 Zurich, Switzerland; 10 Swiss Federal Institute of Technology (ETHZ), 8092 Zurich, Switzerland; 11 Broad Institute of Harvard University and MIT, Cambridge, MA 02142, USA; 12 Department of Biological Engineering, MIT, Cambridge, MA 02139, USA; 13 Department of Brain and Cognitive Sciences, MIT, Cambridge, MA 02139, USA; 14 Center for Neurobiological Engineering, MIT, Cambridge, MA 02139, USA; 15 Harvard-MIT Division of Health Sciences and Technology, MIT, Cambridge, MA 02139, USA

**Keywords:** neuromodulation, electromagnetic, brain, deep brain stimulation, transcranial direct current stimulation, transcranial magnetic stimulation, optogenetics, hippocampus, cortex, noninvasive

## Abstract

We report a noninvasive strategy for electrically stimulating neurons at depth. By delivering to the brain multiple electric fields at frequencies too high to recruit neural firing, but which differ by a frequency within the dynamic range of neural firing, we can electrically stimulate neurons throughout a region where interference between the multiple fields results in a prominent electric field envelope modulated at the difference frequency. We validated this temporal interference (TI) concept via modeling and physics experiments, and verified that neurons in the living mouse brain could follow the electric field envelope. We demonstrate the utility of TI stimulation by stimulating neurons in the hippocampus of living mice without recruiting neurons of the overlying cortex. Finally, we show that by altering the currents delivered to a set of immobile electrodes, we can steerably evoke different motor patterns in living mice.

## Highlights

• Noninvasive TI stimulation electrically stimulates neurons at depth selectively
• Neurons are stimulated by interference between multiple electric fields
• Neurons in mouse hippocampus can be stimulated without affecting the overlying cortex

## Graphical Abstract

**Figure:**
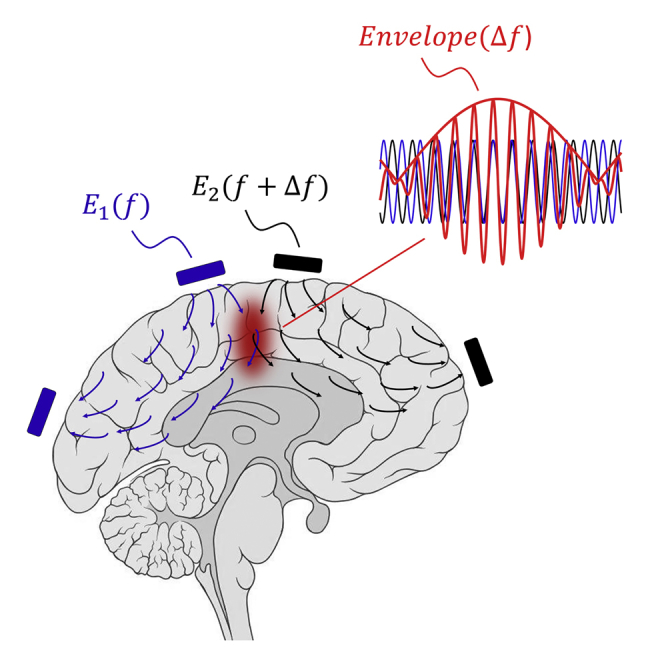

## Teaser

A noninvasive method for deep-brain stimulation may be a new approach for the treatment of neuropsychiatric diseases.

## Introduction

Physical means of brain stimulation, such as the use of implanted electrodes for deep brain stimulation (DBS), have led to widespread excitement about the possibility of repairing neural dysfunction through direct control of brain circuit dynamics, including multiple FDA-approved therapies for previously intractable brain disorders (Greenberg et al., 2010, Kalia et al., 2013). Electrical stimulation via implanted electrodes sparsely activates distributed sets of neurons (Histed et al., 2009), in a fashion different from direct optogenetic control of local cells (Gradinaru et al., 2009). The impact of electromagnetic stimulation on brain circuitry is an emergent function of the fields applied, the excitability properties of the neurons themselves, and the configuration of the neural network in which they are embedded (Merrill et al., 2005). As a result of this complexity, physical means of brain stimulation are often used in a phenomenological way, especially because the excitability properties of neurons vary across different cell types, and thus understanding how a given brain stimulation method impacts a given brain function may require analyzing many factors.

However, some properties of neurons are likely universal—for example, the intrinsic low-pass filtering of electrical signals by the neural membrane (Hutcheon and Yarom, 2000), which prevents neural electrical activity from following very high-frequency oscillating (e.g., ≥ 1 kHz) electric fields. Here, we explore whether the biophysics underpinning such a potentially universal property might support novel strategies for electrical brain stimulation. In particular, if we apply high-frequency oscillating electric fields at multiple sites outside the brain, neurons in the brain will not be able to follow these high-frequency fields directly. However, if two such electric fields are applied at high frequencies that differ by a small amount, which corresponds to a low frequency that neurons can follow, neurons in the brain may be able to demodulate and follow the envelope modulation that results from the temporal interference between these two applied fields, and which oscillates at the difference frequency. If the amplitude of the envelope modulation reaches a maximum at a site deep in the brain, it might be possible to drive deep-lying neurons without recruiting overlying ones. We here test this concept, which we call temporal interference (TI) stimulation, by using computational modeling and phantom measurements, as well as electrophysiological measurements in vivo. We demonstrate the ability of TI stimulation to mediate activation of hippocampal neurons without recruiting overlying cortical neurons and steerably probe motor cortex functionality without physically moving electrodes by altering the current magnitudes delivered to a fixed set of electrodes.

## Results

### TI Stimulation: Concept and Validation of Neural Firing Recruitment

**Figure 1:**
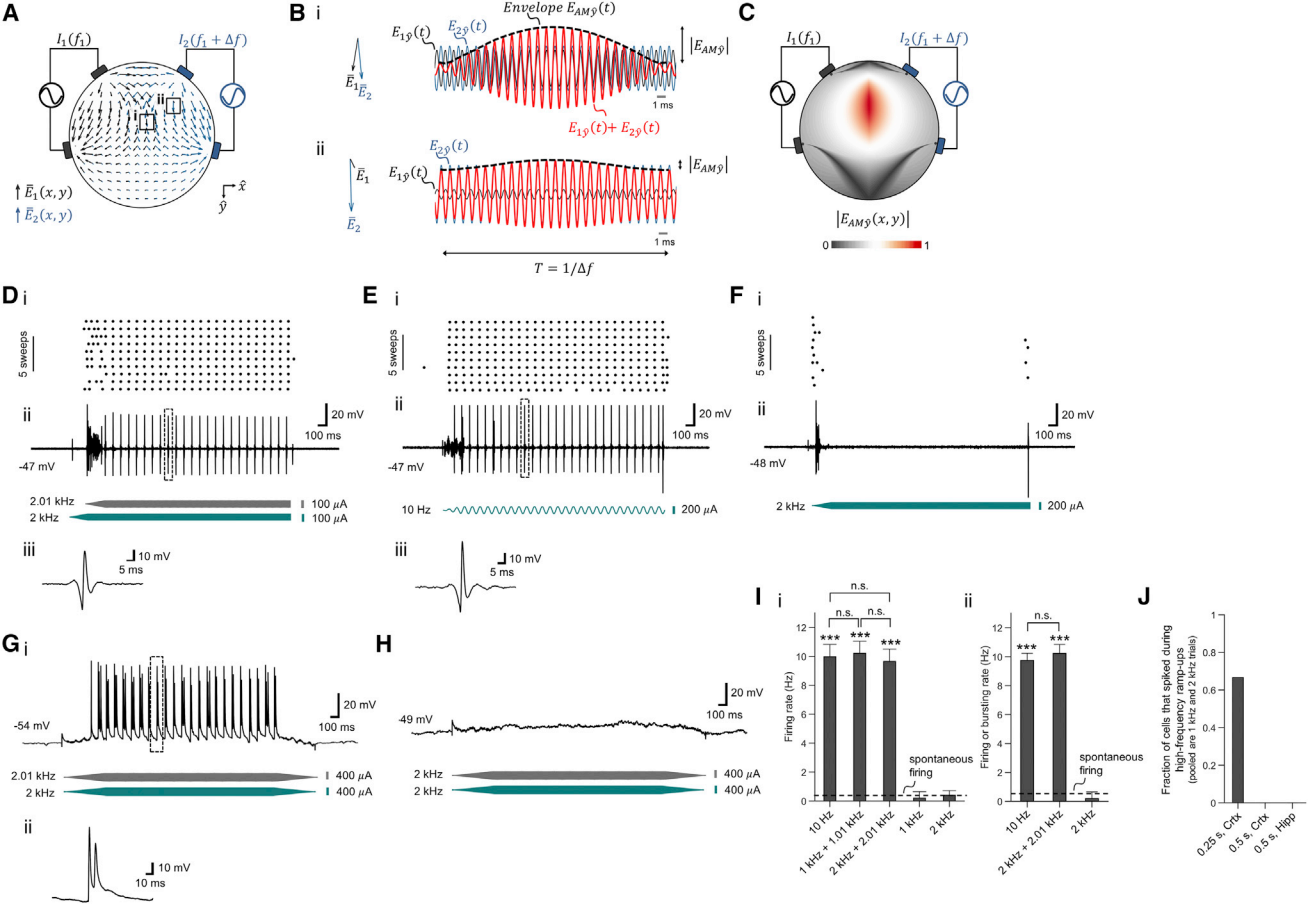
Concept of TI Stimulation and Validation of Neural Activation in Intact Mouse Brain (A–C) TI concept. (A) Electric field vectors ${\bar{E}}_{1}\left(x,y\right)$ and ${\bar{E}}_{2}\left(x,y\right)$ (gray and blue arrows respectively) resulting from alternating currents $I_{1}$ and $I_{2}$ simultaneously applied to the scalp of a simplified head model (simulated as a cylinder filled with saline). $I_{1}$ and $I_{2}$ are applied at kHz frequencies $f_{1}$ (1 mA at 1 kHz in this example, applied across the gray electrodes) and $f_{2}$ (1 mA at 1.04 kHz, across the blue electrodes) that are higher than the range of frequencies of normal neural operation, so that neurons are driven only at the difference frequency. Field amplitudes were normalized to maximum. The field vectors are taken at a time point in which the two currents were applied in-phase from top to bottom electrodes. (B) Magnified views of the electric field vectors ${\bar{E}}_{1}$ and ${\bar{E}}_{2}$ (again normalized to maximum) in the regions indicated by boxes in A and indicated by Roman numerals (*left*), with plots (*right*) of time-domain sinusoidal waveforms of the electric field amplitudes $E_{1\hat{y}}\left(t\right)$ (gray) and $E_{2\hat{y}}\left(t\right)$ (blue) along the $\hat{y}$ direction, as well as the envelope resulting from the superposition of the two fields, i.e., $E_{1\hat{y}}\left(t\right)\;+E_{2\hat{y}}\left(t\right)$ (red). $E_{AM\hat{y}}\left(t\right)$ is the envelope modulation waveform along the $\hat{y}$ direction (black dashed line). (C) Color map (normalized to maximum) of the spatial distribution of the envelope modulation amplitude along the $\hat{y}$ direction (as plotted for two points in B), for the modeled configuration shown in A. (D–J) TI effects on neural activity, assessed with in vivo whole cell patch clamp in anesthetized mouse. (D–F) Representative neural responses from a single patched neuron in the somatosensory cortex undergoing TI stimulation (D) (gray waveform, stimulation at 2.01 kHz, 100 μA amplitude, 0.25 s ramp-up, 1.75 s duration, 0.25 s delay; blue waveform, 2 kHz, 100 μA amplitude, 0.25 s ramp up, 2 s duration, no delay), 10 Hz stimulation (E) (blue waveform, 10 Hz, 200 μA amplitude, 0.25 s ramp-up period, 2 s duration) and high-frequency stimulation (F) (blue waveform, 2 kHz, 200 μA amplitude, 0.25 s ramp-up, 2 s duration). Showing (i) spike raster plots, (ii) traces of current-clamp recording and (iii) magnified views of the trace regions indicated by boxes in (ii). Traces were filtered using a fifth-order Butterworth band-stop filter with cutoff frequencies of 1 kHz and 15 kHz and with a third order Butterworth high-pass filter with a cutoff frequency of 100 Hz to remove 10 Hz and 2 kHz stimulation artifacts; see Figures S1A–S1I for non-filtered traces. (G and H) Representative neural responses from a single patched neuron in hippocampus undergoing TI stimulation (G); gray waveform, stimulation at 2.01 kHz, 400 μA amplitude, 0.5 s ramp-up, 2 s duration, 0.5 s ramp-down; blue waveform, 2 kHz, 400 μA amplitude, 0.5 s ramp up, 2 s duration, 0.5 s ramp-down; shown are (i) traces of current-clamp recording and (ii) magnified views of the trace regions indicated by boxes in (i) and high-frequency stimulation (H); gray waveform, 2 kHz, 400 μA amplitude, 0.5 s ramp-up, 2 s duration, 0.5 s ramp-down; blue waveform, 2 kHz, 400 μA amplitude, 0.5 s ramp-up, 2 s duration, 0.5 s ramp-down). Traces were filtered using a fifth order Butterworth band-stop filter with cutoff frequencies of 1 kHz and 15 kHz to remove 2 kHz stimulation artifacts. (I) Spike frequency in neurons undergoing stimulation, as assessed by whole patch clamp in anesthetized mice (plotted are mean ± SD). (i) Neurons in somatosensory cortex, from left to right: 10 Hz stimulation (200 μA, n = 7 cells from 4 mice), TI stimulation with 1 kHz + 1.01 kHz (current sum 200 μA, n = 6 cells from 2 mice), TI stimulation with 2 kHz + 2.01 kHz (current sum 200 μA, n = 7 cells from 3 mice), 1 kHz stimulation (200 μA, n = 5 cells from 2 mice), 2 kHz stimulation (200 μA, n = 6 cells from 3 mice). (ii) Neurons in hippocampus, from left to right: stimulation with two sinusoids at 10 Hz (current sum 714 ± 367 μA mean ± SD, n = 6 cells from 3 mice), TI stimulation with 2 kHz + 2.01 kHz (current sum 733 ± 100 μA, n = 8 cells from 4 mice), stimulation with two sinusoids at 2 kHz (current sum 880 ± 178 μA, n = 5 cells from 3 mice). Dashed lines, mean spontaneous firing rate; stimulation duration, ∼2 s; ∗∗∗ indicates p < 1.0E-20 for comparison of mean firing rate of a condition versus mean spontaneous firing rate, and n.s. indicates no significant difference between indicated conditions, for post hoc tests following one-way ANOVA with factor of stimulation condition; see Table S1 for full statistics from cortical and hippocampal recordings. See Figures S1J and S1K for traces at different currents for the conditions corresponding to (G)–(H). (J) Fraction of cells that transiently spiked during the high-frequency stimulation ramp-ups (pooled together are 1 kHz with no TI and 2 kHz with no TI); ‘0.25 s, Crtx’, ramp-up period 0.25 s, neurons in cortex, n = 6 cells from 2 mice; ‘0.5 s, Crtx’, ramp-up period 0.5 s, neurons in cortex, n = 6 cells from 3 mice; ‘0.5 s, Hipp’, ramp-up period 0.5 s, neurons in hippocampus, n = 5 cells from 3 mice.

We first set out to examine whether the TI concept could indeed result in well-defined low-frequency envelope modulated electric fields. In the TI concept (Figure 1A), electric currents are applied at high frequencies $f_{1}$ and $f_{2}$ = $f_{1}+\Delta f\;$ that fall outside the range of normal neural operation, but which differ by a small amount, Δf, that falls within the frequency range that neurons can respond to. The superposition of the two electric fields inside the brain results in an electric field at a frequency of $\left(f_{1}+f_{2}\right)/2$, whose envelope is modulated at the frequency Δf (Figure 1B). The amplitude of the envelope modulation at a particular location depends on the vectorial sum of the two applied field vectors at that point and as a result can have a maximum at a point distant from the electrodes, potentially even deep in the brain (Figure 1C). The location of this envelope maximum depends on the electrode configuration, as well as properties of the applied waveforms. For the trapezoidal configuration shown in Figure 1A, the low-frequency envelope oscillates at a frequency of 40 Hz, with waveforms in Figure 1B plotted at the two specific points highlighted by Roman numerals in Figure 1A. For example, Figure 1Bi shows a large envelope modulation amplitude at a location where the two fields are large and aligned, whereas Figure 1Bii shows a small envelope modulation amplitude at a location where the two fields are less aligned.

To assess whether such low-frequency field envelopes could effectively drive neural spiking activity, we applied TI stimulation transcranially to anesthetized living mice, and recorded the responses by using automated whole-cell patch clamp neural recording. Currents were applied via two electrodes on the skull (with a ∼0.5 mm gap between their edges), and recordings were made in the somatosensory cortex. We found that interferential stimulation with two sinusoids at 2.01 kHz and 2 kHz, resulting in a Δf envelope frequency of 10 Hz, was able to recruit neurons to fire at 10 Hz (Figure 1D), as efficaciously as direct 10 Hz stimulation (Figure 1E) that would be expected to broadly affect neural activity (Miranda et al., 2013). High-frequency stimulation (with one sinusoid at 2 kHz and no TI) did not result in activity (Figure 1F), beyond a brief transient associated with the beginning of stimulation in some cells (n = 4 out of 6 cells from 2 mice) when 0.25 s sinusoidal ramp-up times were used. When 0.5 s ramp-up times were used, no such transient activity was observed in any cells (n = 5 cells from 2 mice), suggesting that the transient spiking activity observed earlier was due to the speed of the 0.25 s duration ramp-up (Figure 1J). We validated TI stimulation on a population of cortical cells (Figure 1Ii) and found that interferential stimulation with a difference frequency of 10 Hz resulted in spike frequencies of 10.21 ± 0.83 Hz (mean ± SD), for a 1 kHz carrier frequency (n = 6 cells from 2 mice) and 9.68 ± 0.85 Hz for a 2 kHz carrier frequency (n = 7 cells from 3 mice; see Table S1 for full statistics associated with Figure 1Ii).

To validate whether neuronal firing can be manipulated at different depths in tissue, we performed automatic patch clamp recording in the mouse hippocampus. Currents were applied via two electrodes that were located on the skull, with proximal edges 1.5–2 mm apart. We found that interferential stimulation (with two sinusoids at 2.01 kHz and 2 kHz, resulting in a Δf envelope frequency of 10 Hz) was able to recruit neural firing in synchronization with the envelope—with either single spikes (n = 3 cells from 2 mice) or brief bursts of spikes (n = 5 cells from 3 mice; a burst was defined as a < 50 ms spiking event with inter-spike interval ≤ 15 ms; 1.3 ± 0.37 mean spikes per burst ± SD; 9.07 ± 3.2 ms inter-spike interval) elicited by the TI stimulation (in detail: mouse 1 had one cell with a burst response; mouse 2 had two cells with single spike responses; mouse 3 had two cells with a burst response; mouse 4 had two cells with a burst response and one cell with a single spike response) (Figure 1G). Direct application of high-frequency stimulation (with two sinusoids on the two electrodes, both at 2 kHz) did not result in activity (Figure 1H). No spiking transient was observed because we used the slower, 0.5 s duration ramp-up that we had previously observed to eliminate this transient (Figure 1J; n = 5 cells from 3 mice). We found (Figure 1Iii) that interferential stimulation with a difference frequency of 10 Hz resulted in spike or burst occurrence frequencies of 10.23 ± 0.61 Hz for a 2 kHz carrier frequency (n = 8 cells from 4 mice; see Table S1 for full statistics associated with Figure 1Iii). The timing of the spikes or the first spikes of bursts, relative to the peak of the TI envelope, was −2.8 ± 4.8 ms, i.e., when the envelope amplitude was >97% of its peak amplitude, which was not different from the timing of spikes evoked by 10 Hz stimulation relative to the 10 Hz sinusoid peak (−1.3 ± 2.2 ms; pairwise t test, p = 0.47).

**Figure S1:**
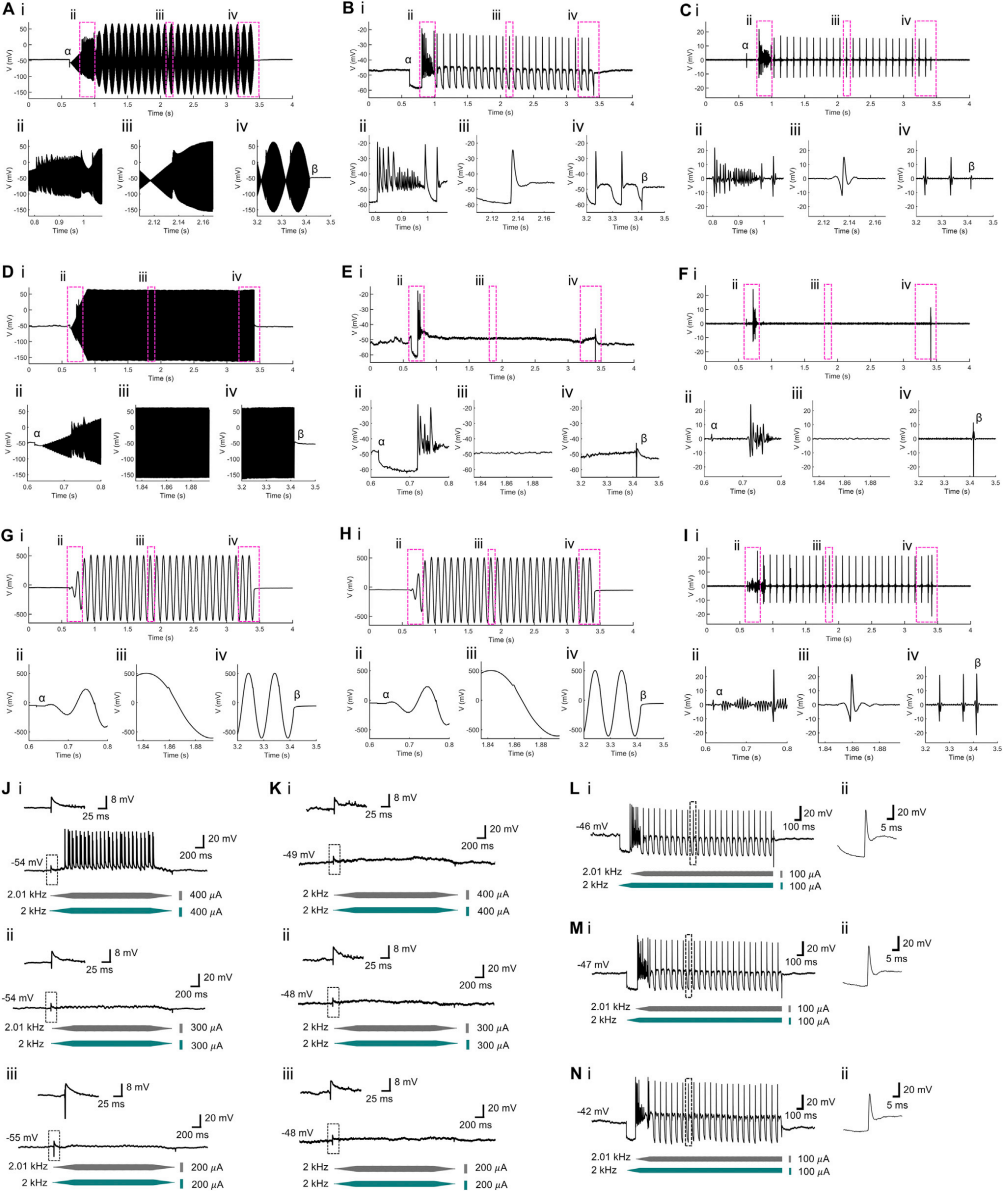
Patch-Clamp Recordings from Cells Undergoing TI Stimulation, Related to Figure 1 (A) to (I) Removal of artifacts from current-clamp recordings as in Figure 1. (i) Trace of current-clamp recording, with (ii–iv) magnified views of the regions indicated by boxes in (i); α, artifact caused by connecting stimulation and recording grounds ($I_{1}=I_{2}=0$ at this point); β, artifact caused by disconnecting stimulation and recording grounds ($I_{1}$ and $I_{2}$ are forced to zero at this point). (A) to (C) TI stimulations as in Figure 1D ($I_{1}$, 2.01 kHz, 100 μA amplitude, 0.25 s ramp-up, 1.75 s duration, 0.25 s delay relative to $I_{2}$; $I_{2}$, 2 kHz, 100 μA amplitude, 0.25 s ramp up, 2 s duration). (A) Raw recording trace. (B) Trace of (A), filtered using a fifth order Butterworth band-stop filter with cutoff frequencies of 1 kHz and 15 kHz. (C) Trace of (B), further filtered using a third order Butterworth high-pass filter with a cutoff frequency of 100 Hz; this is the trace shown in Figure 1D. (D–F) Are as in (A)–(C) but for the case of Figure 1F ($I_{1}$, 2 kHz, 200 μA amplitude, 0.25 s ramp-up, 2 s duration). (G–I) Are as in (A)–(C) but for the case of Figure 1E ($I_{1}$, 10 Hz, 200 μA amplitude, 0.25 s ramp-up period, 2 s duration); ringing in (Iii) is filtering distortion due to the Gibbs phenomenon. (J and K) Representative neural responses from a single patched neuron in the hippocampus, the neuron of Figures 1G and 1H, undergoing TI stimulation (J); gray waveform, stimulation at 2.01 kHz; blue waveform, 2 kHz) or high-frequency stimulation (K); gray waveform, 2 kHz; blue waveform, 2 kHz) with current amplitude of (i) 400 μA; (ii) 300 μA; (iii) 200 μA. The stimulation order was (iii), (ii), (i) with 2 s intervals between consecutive stimulations. Trace regions containing artifacts caused by connecting stimulation and recording devices (i.e., before current amplitudes are ramped up) are indicated by boxes, with magnified views shown above the boxes. (L–N) Representative neural responses from a single patched neuron in the anesthetized mouse somatosensory cortex undergoing repeated TI stimulation (gray waveform, stimulation at 2.01 kHz, 100 μA amplitude, 0.25 s ramp-up, 1.75 s duration, 0.25 s delay relative to blue waveform; blue waveform, 2 kHz, 100 μA amplitude, 0.25 s ramp up, 2 s duration, no delay) with 2 s intervals between repetitions. (i) Neural response trace, (ii) magnified view of region indicated by a box in (i). (L) Representative trace from the first stimulation period. (M) Representative trace from the 10th stimulation period. (N) Representative trace from the 20th stimulation period. To remove stimulation artifacts, all traces in the figure were filtered using a fifth order Butterworth band-stop filter with cutoff frequencies of 1 kHz and 15 kHz.

The membrane potential of neurons undergoing TI stimulation repolarized between single spikes, or between brief bursts of spikes, to the baseline membrane potential (cortex, −10.36 ± 27.84 mV, mean difference from baseline ± SD; p = 0.74, pairwise t test; n = 13 cells from 5 mice; hippocampus, 5.5 ± 7.89 mV; p = 0.34; n = 8 cells from 4 mice). The spike frequency during the 20th bout of TI stimulation (tested in three cells in the somatosensory cortex from one mouse; 2 s stimulation followed by 2 s rest) was 9.93 ± 0.2 Hz (mean ± SD), not different from the spike frequency during the 1st bout (p = 0.95; pairwise t test), and the spike amplitude during the 20^th^ bout of TI stimulation was not different from the spike amplitude during the first bout (5.3 ± 3.5 mV, mean amplitude difference ± SD; p = 0.75, pairwise t test); see Figures S1L–S1N for representative traces. The membrane potential of neurons undergoing high-frequency stimulation (with two sinusoids on the two electrodes, both at 2 kHz or at 1 kHz) in both the cortex and the hippocampus was not different from the baseline membrane potential before the stimulation (cortex, 1.67 ± 4.87 mV, mean difference from baseline ± SD, measured 1 s after stimulation onset; p = 0.66, pairwise t test; n = 11 cells from 5 mice; hippocampus, −1.7 ± 5.39 mV; p = 0.91; n = 5 cells from 3 mice).

### Validation of Steerability Using Computational Modeling and Tissue Phantom

**Figure S2:**
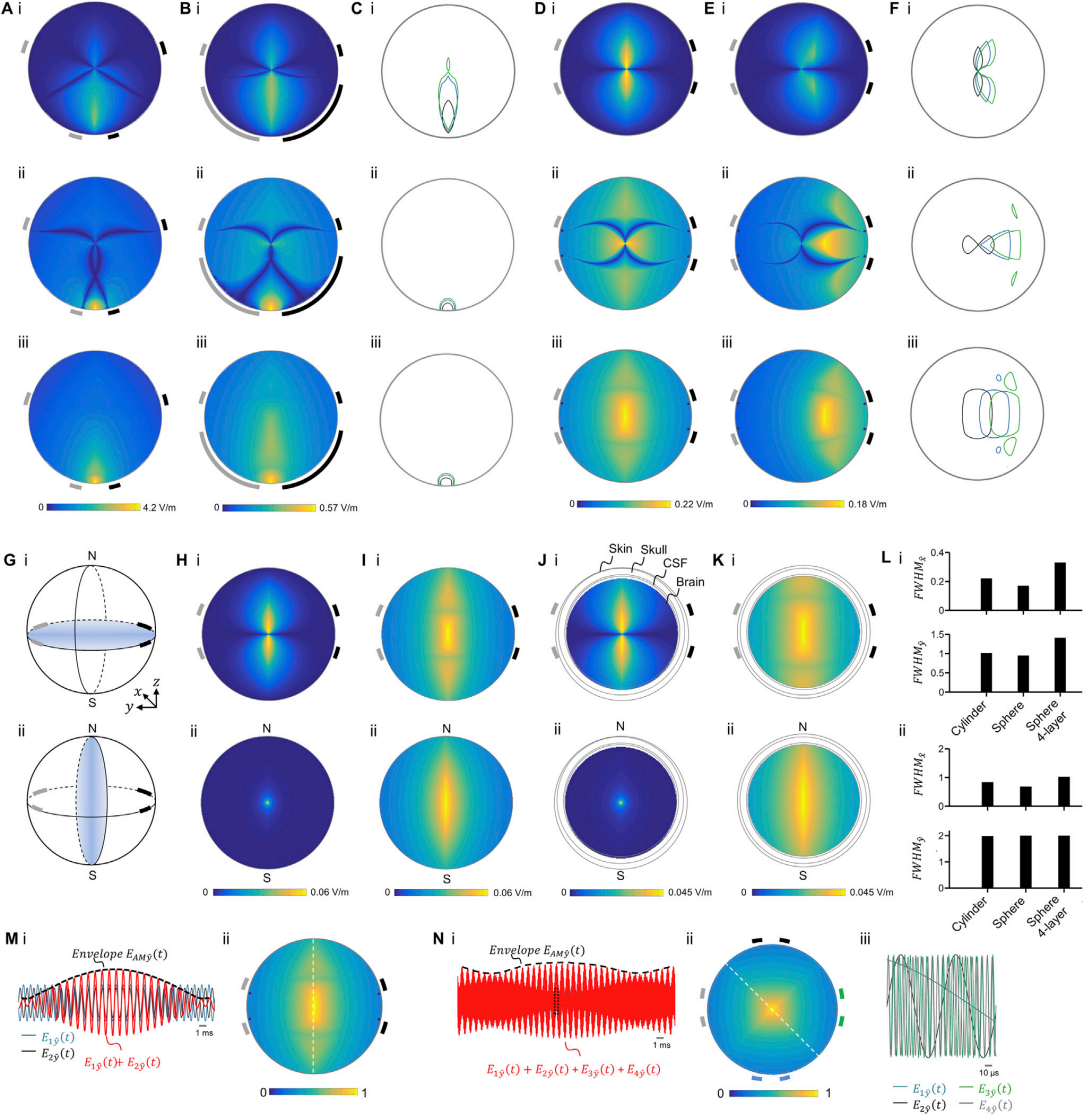
Simulation of TI Fields in a Phantom, Related to Figure 2 An alternating current $I_{1}$ was applied to a phantom via one pair of surface electrodes (gray) at a ∼kHz frequency, $f_{1}$. A second alternating current $I_{2}$ was simultaneously applied to the phantom via a second pair of surface electrodes (black) at a ∼kHz frequency $f_{2}=f_{1}+\Delta f$ where $\Delta f\ll f_{1}$. The electrodes were electrically isolated. The spatial distributions of the electric fields ${\bar{E}}_{1}$ and ${\bar{E}}_{2}$, from currents $I_{1}$ and $I_{2}$ respectively, were simulated independently using a finite element method. The spatial distribution of the envelope modulation amplitude from the superposition of ${\bar{E}}_{1}$ and ${\bar{E}}_{2}$ was computed for a projection direction radial to the surface of the phantom, i.e., $\left|E_{AM\hat{r}}\left(x,y\right)\right|$, and for a projection direction tangential to the surface of the phantom, i.e., $\left|E_{AM\hat{t}}\left(x,y\right)\right|$, using $\left|{\vec{E}}_{AM}\left(\vec{n,}x,y\right)\right|=\left|\left|\left({\vec{E}}_{1}\left(x,y\right)+{\vec{E}}_{2}\left(x,y\right)\right)\cdot \vec{n}\right|-\left|\left({\vec{E}}_{1}\left(x,y\right)-{\vec{E}}_{2}\left(x,y\right)\right)\cdot \vec{n}\right|\;\right|$, where $\vec{n}$ is a unit vector in radial or tangential direction. The maximal envelope amplitude $\left|E_{AM}^{max}\left(x,y\right)\right|$ that was generated by the vector fields ${\vec{E}}_{1}$ and ${\vec{E}}_{2}$ at locations (x,y) across all directions was computed in post-processing as described in the STAR Methods. (A–F) Cylindrical phantom model. The phantom model was a cylinder with a 50 mm diameter and 10 mm height that was filled with a saline solution (conductivity 0.333S/m). Figure panels show (i) envelope modulation amplitude $\left|E_{AM\hat{r}}\left(x,y\right)\right|$, (ii) envelope modulation amplitude $\left|E_{AM\hat{t}}\left(x,y\right)\right|$ and (iii) maximal envelope modulation amplitude along any direction $\left|E_{AM}^{max}\left(x,y\right)\right|$. Color-maps are in units of V/m. Distances were normalized to the phantom’s radius. (A–C) The volume targeted for large envelope modulation amplitude is largely independent of electrode size. (A) Envelope modulation amplitude maps. The two pairs of electrodes (gray and black) were placed in an isosceles trapezoid geometry such that each electrode pair was located at the vertices of one lateral side. The trapezoid had a normalized small base size of a = 0.39 and a normalized large base size of b = 1.96 (geometry as in Figure 2A). The amplitudes of currents $I_{1}$ and $I_{2}$ were 1 mA. (B) As (A) but with approximately 8× larger electrodes (normalized electrode size of 1.3) at the vertices of the lower base while holding the space between the edges of these two electrodes fixed. (C) Contours of 1/e of the peak value of the envelope modulation amplitude. Electrodes at the small trapezoid base had a normalized size of 0.16 (black; corresponding to envelope modulation maps in (A)), 0.5 (blue), and 1.3 (green; corresponding to envelope modulation maps in (B)). (D–F) Steering of the large envelope modulation volume between two pairs of fixed electrodes. (D) Envelope modulation amplitude maps. The two pairs of electrodes (gray and black) were placed at a rectangular geometry with a normalized length of 1.96 (geometry as in Figure 2C). The amplitudes of currents $I_{1}$ and $I_{2}$ were 1 mA as in (A). (E) As (D) but current $I_{1}$ between the gray electrode pair was increased by ΔI=0.6mA and current $I_{2}$ between the black electrode pair was decreased by the equal ΔI (i.e., total current $I_{1}+I_{2}$ was not changed), so that the current ratio $I_{1}\text{:}I_{2}$ was 4:1. (F) Contours of 1/e of the peak value of the envelope modulation amplitude. Current ratio $I_{1}:I_{2}$ was 1:1 (black; corresponding to envelope modulation maps in (D)), 2.5:1 (blue), and 4:1 (green; corresponding to envelope modulation maps in (E). (G) to (L) Spherical phantom model. The phantom model was a conductive sphere with a 50 mm diameter. The electrodes were arranged in a rectangular geometry with a normalized length of 1.96 (geometry as in Figure 2C). Panels (H) to (K) show envelope modulation amplitude distributions in (i) the electrode plane and (ii) a plane perpendicular to the plane of the electrodes as schematized in (G) (N and S indicate the north and south poles of the sphere, respectively). Color-maps are in units of V/m. Distances were normalized to phantom radius. (G) Schematic illustration of the phantom model showing (i) in-plane and (ii) a perpendicular plane with respect to the plane of the electrodes. (H and I) Sphere with homogeneous conductivity of 0.333S/m. (H) Envelope modulation amplitude maps of $\left|E_{AM\hat{r}}\left(x,y\right)\right|$. (I) Envelope modulation amplitude maps of $\left|E_{AM}^{max}\left(x,y\right)\right|$. (J and K) Sphere with inhomogeneous conductivity consisting of 4 layers: scalp (d=0.05, σ=0.333S/m), skull (d=0.085, σ=0.0083S/m), cerebrospinal fluid (d=0.023, σ=1.79S/m) and brain (d=0.83, σ=0.333S/m), where d is the normalized layer thickness. (J) Envelope modulation amplitude maps of $\left|E_{AM\hat{r}}\left(x,y\right)\right|$. (K) Envelope modulation amplitude maps of $\left|E_{AM}^{max}\left(x,y\right)\right|$. (L) Comparison of normalized full width at half maxima (FWHM) of envelope modulation amplitude maps (i) $\left|E_{AM\hat{r}}\left(x,y\right)\right|$ and (ii) $\left|E_{AM}^{max}\left(x,y\right)\right|$ in the plane of the electrodes when the phantom was: a homogeneous cylindrical plate (‘cylinder’; panels (A) to (F)), homogeneous sphere (‘sphere’; panels (H) and (I)) and inhomogeneous 4-layer sphere (‘sphere 4-layer’; panels (J) and (K)) of equal diameter (50 mm). $FWHM_{\hat{x}}$ and $FWHM_{\hat{y}}$ are FWHM along $\hat{x}$ and $\hat{y}$ directions, respectively. (M and N) Cylindrical phantom model with different number of fields. n alternating currents $\left\{I_{1},I_{2},\ldots ,I_{n}\right\}$ at different kHz frequencies $\left\{f_{1},f_{2},\ldots ,f_{n}\right\}$ were applied simultaneously to a phantom (as in Figure 2) via n pairs of surface electrodes. Electrode pairs were placed at the circumference with equal spacing and applied currents of 1 mA. Shown is (i) a time-domain plot of sinusoidal waveforms of the electric field amplitudes $\left\{E_{1\hat{y}}\left(t\right),\;E_{2\hat{y}}\left(t\right),\ldots ,E_{n\hat{y}}\left(t\right)\right\}$ along the $\hat{y}$ direction, as well as the waveform resulting from the superposition of the fields, i.e., $\sum E_{1\hat{y}}\left(t\right),\;E_{2\hat{y}}\left(t\right),\ldots ,E_{n\hat{y}}\left(t\right)$ (red). $E_{AM\hat{y}}\left(t\right)$ is the envelope of the superposition waveform along the $\hat{y}$ direction (black dashed line). Shown in (ii) is the maximal envelope amplitude across all directions $\left|E_{AM}^{max}\left(x,y\right)\right|$; color-maps are values normalized to the maximum value. (M) TI fields with n=2 alternating currents $\left\{I_{1},I_{2}\right\}$ applied via electrode pairs {gray, black} at frequencies $\left\{f_{1}=1\;kHz,f_{2}=1.04\;kHz\right\}$. Panel (i) is as in Figure 1Bi and panel (ii) is as in Diii, replotted here for comparison with TI fields with n=4. Half width half maximum (HWHM) of the main peak normalized to the phantom radius is 0.49, computed along the white dashed line. (N) TI fields with n=4 alternating currents $\left\{I_{1},I_{2},I_{3},I_{4}\right\}$ applied via electrode pairs {blue, black, green, gray} at frequencies {*f*_1_ = 1.04 *kHz*, *f*_2_ = 9 *kHz*, *f*_3_ = 90 *kHz*, *f*_4_ = 100 *kHz*}. The maximal envelope amplitude $\left|E_{AM}^{max}\left(x,y\right)\right|$ that was generated by n>2 vector fields was approximated using $2\cdot min\left\{{\vec{E}}_{1}\left(\vec{r}\right),{\vec{E}}_{2}\left(\vec{r}\right),\ldots ,{\vec{E}}_{n}\left(\vec{r}\right)\right\}$. HWHM of the main peak normalized to the phantom radius is 0.23, computed along the white dashed line. (iii) Magnified view of the boxed region in (i), plotted without the superposition waveform.

**Figure S3:**
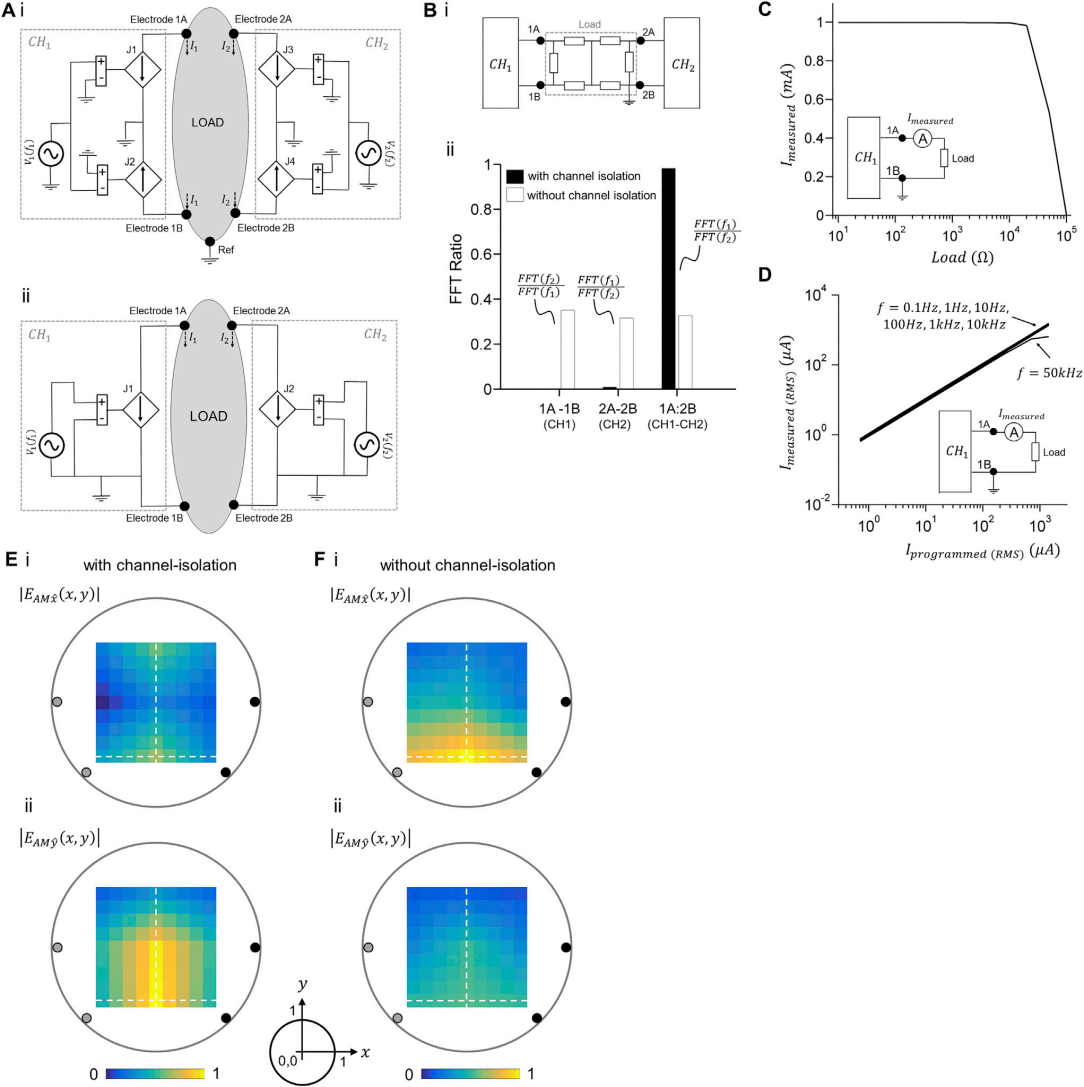
Design, Implementation, and Characterization of TI Stimulator, Related to Figure 2, Figure 3, Figure 4, Figure 5 and STAR Methods Stimulating currents were generated using a custom device consisting of two electrically isolated current sources. To isolate the channels, each waveform was supplied via a balanced pair of current sources that were driven in precisely opposite phase, a technique that we call anti-phasic current drive. (A) Schematics of the electronic circuitry of the stimulator. (i) Dual channel stimulation with anti-phasic current drive isolation. In channel 1 $\left(CH_{1}\right)$, a voltage waveform $V_{1}$ at a frequency $f_{1}$ was applied to the positive (+) input of a voltage-controlled current source (J1) that had its negative (−) input grounded, resulting in a current waveform $I_{1}$ at node 1A that was in-phase with waveform $V_{1}.$ An equal voltage waveform $V_{1}$ at a frequency $f_{1}$ was applied to the negative (−) input of a second voltage-controlled current source (J2) that had its positive (+) input grounded, resulting in a current waveform $-I_{1}$ at node 1B that is anti-phase with waveform $V_{1}.$ In channel 2 $\left(CH_{2}\right)$, a second voltage waveform $V_{2}$ at a frequency $f_{2}$ was converted in an equivalent way to an in-phase current waveform $I_{2}$ at node 2A by a voltage-controlled current source (J3) and to an anti-phase current waveform $-I_{2}$ at node 2B by a voltage-controlled current source (J4). The amplitude of current $I_{1}$ of $CH_{1}$ between nodes 1A and 1B was calibrated such that $I_{1}\left(A\right)=\left(V_{1}\left(V\right)\;/500\right)$ and the amplitude of current $I_{2}$ of CH2 between nodes 2A and 2B was calibrated such that $I_{2}\left(A\right)=\left(\;V_{2}\left(V\right)/500\right)$. A ground or reference electrode (Ref) was provided to carry any imbalance currents from the paired current sources and to prevent charging of the body relative to earth ground. (ii) Dual channel stimulation without isolation. As in (i), but nodes 1B and 2B were connected to the GND of the device. (B) Characterization of channel isolation. (i) Schematic of the experiment setup. Voltage waveform $V_{1}$ of $CH_{1}$ was set to 1 kHz and 0.5 V resulting in a current $I_{1}$ between nodes 1A and 1B at the same frequency and an amplitude of 1 mA. The output nodes 1A and 1B were connected to a load made of a bridge of 6 resistors with 1 kΩ resistance each. Voltage waveform $V_{2}$ of $CH_{2}$ was set to 1.1 kHz and 0.5 V resulting in a current $I_{2}$ between nodes 2A and 2B at the same frequency and an amplitude of 1 mA. The output nodes 2A and 2B were connected to the same resistor bridge load as shown in the schematics. The frequency spectrum of the currents was measured using a FFT spectrum analyzer (SR770, Stanford Research) at the output of $CH_{1}$ between nodes 1A and 1B, the output of $CH_{2}$ between nodes 2A and 2B, and across the resistor bridge between nodes 1A and 2B. (ii) Ratio of the FFT amplitude at the cross-talk frequency (i.e., $f_{2}$ at the output nodes 1A and 1B of $CH_{1}$ and $f_{1}$ at the output nodes 2A and 2B of $CH_{2}$) and the FFT amplitude at the channel’s set frequency (i.e., $f_{1}$ at the output nodes 1A and 1B of $CH_{1}$ and $f_{2}$ at the output nodes 2A and 2B of $CH_{2}$). FFT ratio across $CH_{1}-CH_{2}$ between the output node 1A of $CH_{1}$ and the output node 2B of $CH_{2}$ is the ratio of the FFT amplitude of $f_{1}$ and the FFT amplitude of $f_{2}$. (The total harmonic distortion of the current source was < 0.08% at 100 Hz and < 0.4% at 10 kHz, measured with 9 harmonics on 1 kΩ load resistor.) (C) Characterization of output current for different load resistances. Voltage waveform $V_{1}$ of $CH_{1}$ was set to 1kHz and 0.5 V resulting in a current $I_{1}$ between nodes 1A and 1B of the same frequency and an amplitude of 1 mA. The output nodes 1A and 1B were connected to loads with resistances between 100 Ω and 100 kΩ. The output nodes 2A and 2B of $CH_{2}$ were grounded. The current flowing between nodes 1A and 1B was measured using a digital ammeter. The panel shows the amplitude of the measured currents $I_{measured}$ in mA against the load resistance in Ω. (D) Characterization of output current for different set frequencies. Voltage waveform $V_{1}$ of $CH_{1}$ was set to a range of frequencies between 0.1 Hz and 50 kHz with a range of amplitudes between 0.5 mV and 0.5 V, resulting in a current $I_{1}$ between 1A and 1B nodes of the same frequencies and with amplitudes that ranged between 1 μA and 1 mA. The output nodes 1A and 1B were connected to a load with a resistance of 10 kΩ. The output nodes 2A and 2B of $CH_{2}$ were grounded. The current flowing between nodes 1A and 1B was measured using a digital ammeter. The panel shows 7 line plots of the RMS amplitude of the measured currents $I_{measured\;\left(RMS\right)}$ in μA against the RMS amplitude of the current that was programmed in the device $I_{programmed\;\left(RMS\right)}$ in μA, where $I_{programmed\;\left(RMS\right)}=\left(I_{1}/\sqrt{2}\right)=\left(V_{1}\left(V\right)/\sqrt{2}\cdot 500\;\right)$, for frequencies 0.1 Hz, 1 Hz, 10 Hz, 100 Hz, 1kHz, 10 kHz and 50 kHz. (Note that the line plots of frequencies between 0.1 Hz and 10 kHz are overlapping). (E and F) Effect of channel isolation on distribution of envelope amplitude. An alternating current $I_{1}$ was applied to a phantom at a frequency of 1 kHz via one pair of electrodes (gray). A second alternating current $I_{2}$ was applied to the phantom at a frequency of 1.02 kHz via a second pair of electrodes (black). The phantom was a non-conductive cylinder of 50 mm diameter and 10 mm height that was filled with a saline solution. The two pairs of electrodes (gray and black) were placed in an isosceles trapezoid geometry such that each electrode pair was located at the vertices of one lateral side. The trapezoid had a normalized small base size of a = 1.39 and a normalized large base size of b = 1.96. The amplitudes of currents $I_{1}$ and $I_{2}$ were 1 mA. The envelope modulation amplitude from temporal interference of two electric fields projected along the $\hat{x}$ and $\hat{y}$ directions was measured using a lock-in amplifier as in Figure 2 (see also STAR Methods for a detailed description of the phantom measurement). Envelope modulation amplitude maps are a linear interpolation (interpolation factor 2) between the measured values. Color-maps show values normalized to maximal envelope modulation amplitude. Distances were normalized to the phantom’s radius and are shown relative to the center of the phantom. High isolation is required between the two current sources in order to focus the region with large envelope modulation amplitude deep into the phantom. (E) Envelope modulation amplitude maps when currents were applied with a high level of electrical isolation between the current sources. (i) Envelope modulation amplitude map $\left|E_{AM\hat{x}}\left(x,y\right)\right|$ along $\hat{x}$ direction; (ii) envelope modulation amplitude map $\left|E_{AM\hat{y}}\left(x,y\right)\right|$ (projection along $\hat{y}$ direction). Dashed lines cross at the peak of the envelope modulation amplitude distribution, i.e., ${\left|E_{AM\hat{x}}\right|}_{max}$ and ${\left|E_{AM\hat{y}}\right|}_{max}$. The volume of large envelope modulation amplitude was located along the midline of trapezoid at its small base with a peak at x = 0 and y = 0.49. The spread of $\left|E_{AM\hat{x}}\right|$ around its peak has a normalized half width at half maximum along the $\hat{x}$ direction $HWHM_{\hat{x}}=0.46$ and a normalized half width at half maximum along the $\hat{y}$ direction $HWHM_{\hat{y}}=0.46$. The spread of $\left|E_{AM\hat{y}}\right|$ around its peak is $HWHM_{\hat{x}}=0.51$ and $HWHM_{\hat{y}}=0.95$. (F) Same as (E) but when currents were applied without electrical isolation between the current sources. The peak of the envelope modulation amplitude was located along the midline of the trapezoid at its small base as in (E) however the distribution of the envelope modulation amplitude was significantly more dispersed. The amplitude of $\left|E_{AM\hat{x}}\right|$ at the end of the $\hat{x}$ direction dashed line (normalized distance of 0.51 from center) was 0.76 of its maximal value. The spread of $\left|E_{AM\hat{x}}\right|$ around its maximal value had a normalized $HWHM_{\hat{x}}=0.95$. The amplitude of $\left|E_{AM\hat{y}}\right|$ at the end of the $\hat{y}$ direction dashed line was 0.91 of its maximal value. The spread of $\left|E_{AM\hat{y}}\right|$ around its maximal value had a normalized $HWHM_{\hat{y}}=0.83$.

**Figure 2:**
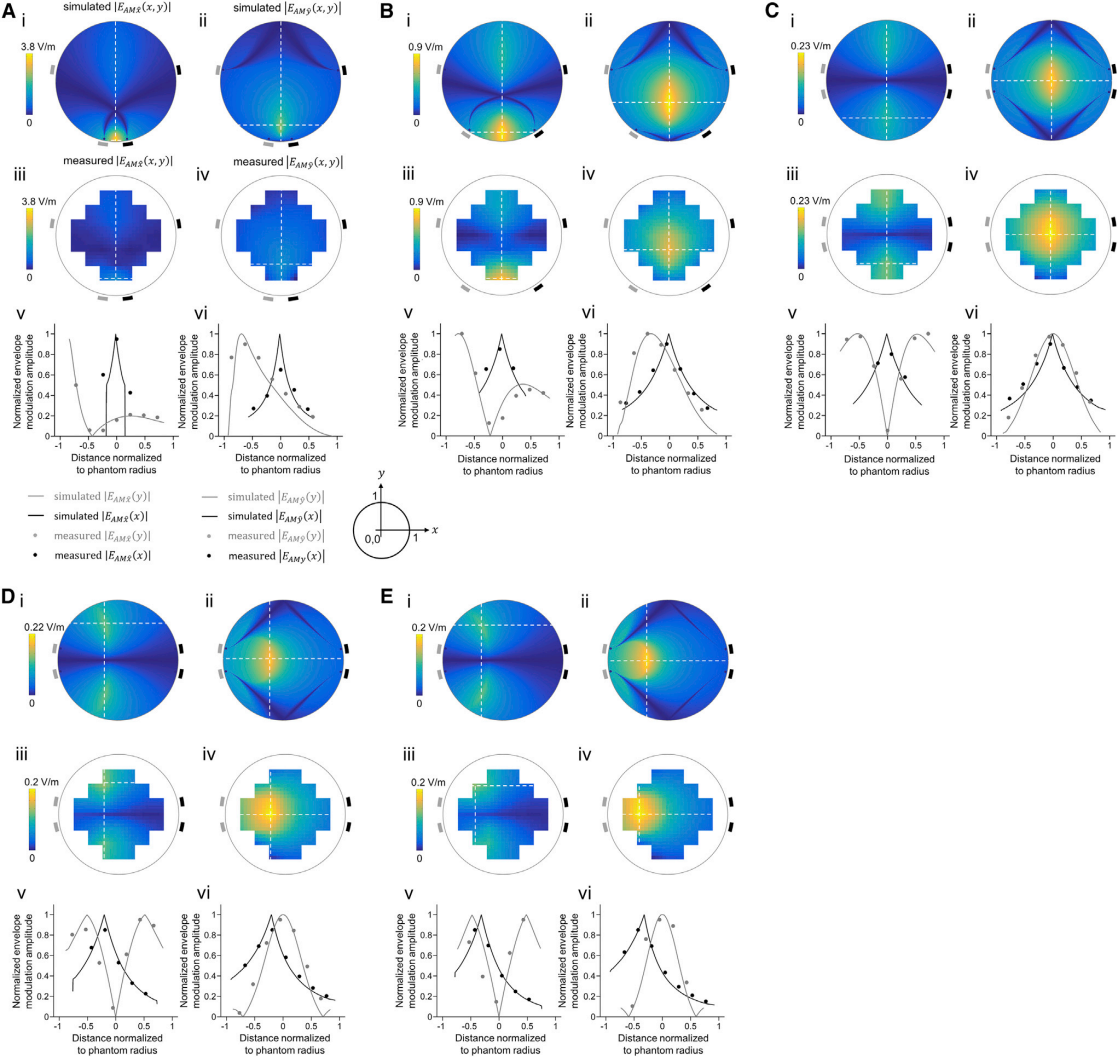
Steerability of TI, Probed via Both Computational Modeling and a Tissue Phantom For each condition (A)–(E), we simulated the interferential electric field envelope modulation (projected along: i, x-direction, ii, y-direction) that would result from electrodes at the locations indicated by the rectangles (the gray electrodes forming a pair, with an alternating current $I_{1}$ applied at 1 kHz, and the black electrodes forming a second pair, with an alternating current $I_{2}$ applied at 1.02 kHz), passing the currents described below in the individual panel caption sections. For exact coordinates of electrodes and numerical values of the peak envelope modulation amplitude, location, and width, see Table 1. We also experimentally measured in a tissue phantom (a non-conductive cylinder of 50 mm diameter and 10 mm height that was filled with a saline solution, with 1 mm diameter silver wire electrodes at various points around the perimeter of the phantom) these two amplitudes (iii, x-direction, iv, y-direction); channels were isolated as described in Figure S3. Finally, we plotted, along line cuts through the simulated (lines) and experimental (dots) datasets, the interferential electric field envelope amplitudes for the x-direction (v) and the y-direction (vi). Simulated and experimental values along the vertical line cut were plotted in gray, and along the horizontal line cut, in black; values were normalized to the peak. Color-maps in i-iv are in V/m. Envelope modulation amplitude maps in iii and iv are a linear interpolation of the measured values. Distances in v and vi were normalized to the phantom’s radius and shown relative to the center of the phantom. Circles in line plots v and vi are measured envelope modulation amplitudes without interpolation. (A) Electrodes were placed in a trapezoidal geometry with a narrow base, and amplitudes of currents $I_{1}$ and $I_{2}$ were set to 1 mA. (B) Electrodes were placed in a trapezoidal geometry with a wider base, with currents as in (A). (C) Electrodes were placed in a rectangle, with currents as in (A). (D) Electrodes as in (C), but now with currents in the ratio $I_{1}:I_{2}=1:2.5$ (holding the sum at 2 mA). (E) Electrodes as in (C), but now with currents in the ratio $I_{1}:I_{2}=1:4$ (holding the sum at 2 mA).

To explore the effects of interferential stimulation at a physics level, we modeled the interferential electric field envelope magnitude as in Figure 1A but for a variety of electrode configurations, and we also experimentally assessed these fields by using a tissue phantom comprising a plastic cylinder filled with saline (Figure S2). Current sources were isolated in the circuitry as described in Figure S3. We found that by altering the locations of the electrodes and by setting the currents appropriately, we could enable the interferential envelope modulation to be targeted to specific locations. For example, when the electrodes were arranged in a trapezoidal geometry as in Figure 1A, but with a narrow base, we obtained, both in simulation and in the phantom, a peak of envelope modulation near the surface of the cylinder, at a point in between the two electrodes (Figure 2A), when both electrode pairs were conducting equal currents (1 mA in this example). By moving the electrodes that comprise the narrow base of the trapezoid further and further apart from each other, holding the currents constant, we could steer the location of the peak envelope modulation deeper into the tissue (Figure 2B), approaching the center of the cylinder as the trapezoid converged to a rectangle (Figure 2C). The envelope locus (i.e., distance out to 1/e of the envelope maximum) in Figure 2C was approximately two times larger, and the peak envelope amplitude ten times weaker, than in Figure 2A (see Table 1 for numerical values associated with these three panels). Thus, it is possible to steer the envelope peak to have its maximum at essentially any depth throughout a volume, albeit with a tradeoff between the locus depth and its width and strength. Varying the locations of electrodes causes large changes in steering, with electrode size variation playing a more minor role (Figures S2A–S2C). These analyses were conducted with a cylindrical phantom, but similar field distributions were obtained in simulations with a spherical phantom (Figures S2G–S2L).

**Table 1:** Summary of Envelope Amplitude Attributes in Cylindrical Phantom

Figure 2 Panel	Electrodes’ Trapezoid		Current Ratio (Gray to Black)	Envelope Modulation Amplitude ${\left|E_{AM\hat{x}}\right|}_{max}$			Envelope Modulation Amplitude ${\left|E_{AM\hat{y}}\right|}_{max}$		
	Bottom Base	Top Base		Peak (V/m) (Simulated, Measured)	Normalized Peak Location (x,y)	Normalized Half Width Half Maximum ($HWHM_{\hat{x}},$$HWHM_{\hat{y}}$)	Peak (V/m) (Simulated, Measured)	Normalized Peak Location (x,y)	Normalized Half Width Half Maximum ($HWHM_{\hat{x}}$, $HWHM_{\hat{y}}$)
A	0.39	1.96	1:1	(3.7, 1)	(0, −1)	(0.18, 0.1)	(2.5, 1.1)	(0, −0.77)	(0.18, 0.37)
B	1.11	1.96	1:1	(0.9, 0.75)	(0, −0.87)	(0.33, 0.25)	(0.9, 0.7)	(0, −0.37)	(0.4, 0.52)
C	1.96	1.96	1:1	(0.13, 0.15)	(0, −0.59)	(0.37, 0.42)	(0.21, 0.23)	(0, 0)	(0.42, 0.49)
D	1.96	1.96	1:2.5	(0.13, 0.14)	(−0.2, −0.59)	(0.37, 0.42)	(0.2, 0.22)	(−0.2, 0)	(0.38, 0.4)
E	1.96	1.96	1:4	(0.12, 0.13)	(−0.35, 0)	(0.36, 0.42)	(0.19, 0.2)	(−0.35, 0)	(0.37, 0.34)

Distances were normalized to the phantom’s radius, with (0, 0) at the center of the phantom. ${\left|E_{AM\hat{x}}\right|}_{max}$ and ${\left|E_{AM\hat{y}}\right|}_{max}$ are maximum values of the envelope modulation amplitude along $\hat{x}$ and $\hat{y}$ directions, respectively. $HWHM_{\hat{x}}$ and $HWHM_{\hat{y}}$ are normalized half width at half maximum along $\hat{x}$ and $\hat{y}$ directions, respectively. Current ratio is the ratio between the current applied by the gray electrode pair and the current applied by the black electrode pair (the scalar sum was set to 2 mA).

We next explored whether tuning the currents, while holding electrode locations constant, could be used to control the field envelope locus, and in particular to steer the envelope modulation peak away from the centroid of the electrode locations. We started by taking the electrode configuration of Figure 2C, with its rectangular geometry, and adjusting the ratio of currents across the gray versus black electrodes from 1:1 to 1:2.5 (Figure 2D) and 1:4 (Figure 2E). We found that by changing the current ratio between the electrode pairs, while keeping the current sum fixed, the peak envelope modulation became increasingly close to the electrode pair with the lower current, with the peak moving 20% of the radius away from the center in the 1:2.5 case (Figure 2D) and 35% of the radius away from the center in the 1:4 case (Figure 2E). This suggests the possibility of “live steering” of activity from one deep site to another within the brain, without having to physically move the electrodes themselves. By having a larger number of electrodes on the scalp, and tuning the current frequencies and amplitudes appropriately, it may be possible to make the deep targeted stimulation volume smaller, as we computationally model in Figures S2M and S2N.

### Stimulation of Mouse Hippocampus but Not Overlying Cortex

**Figure S4:**
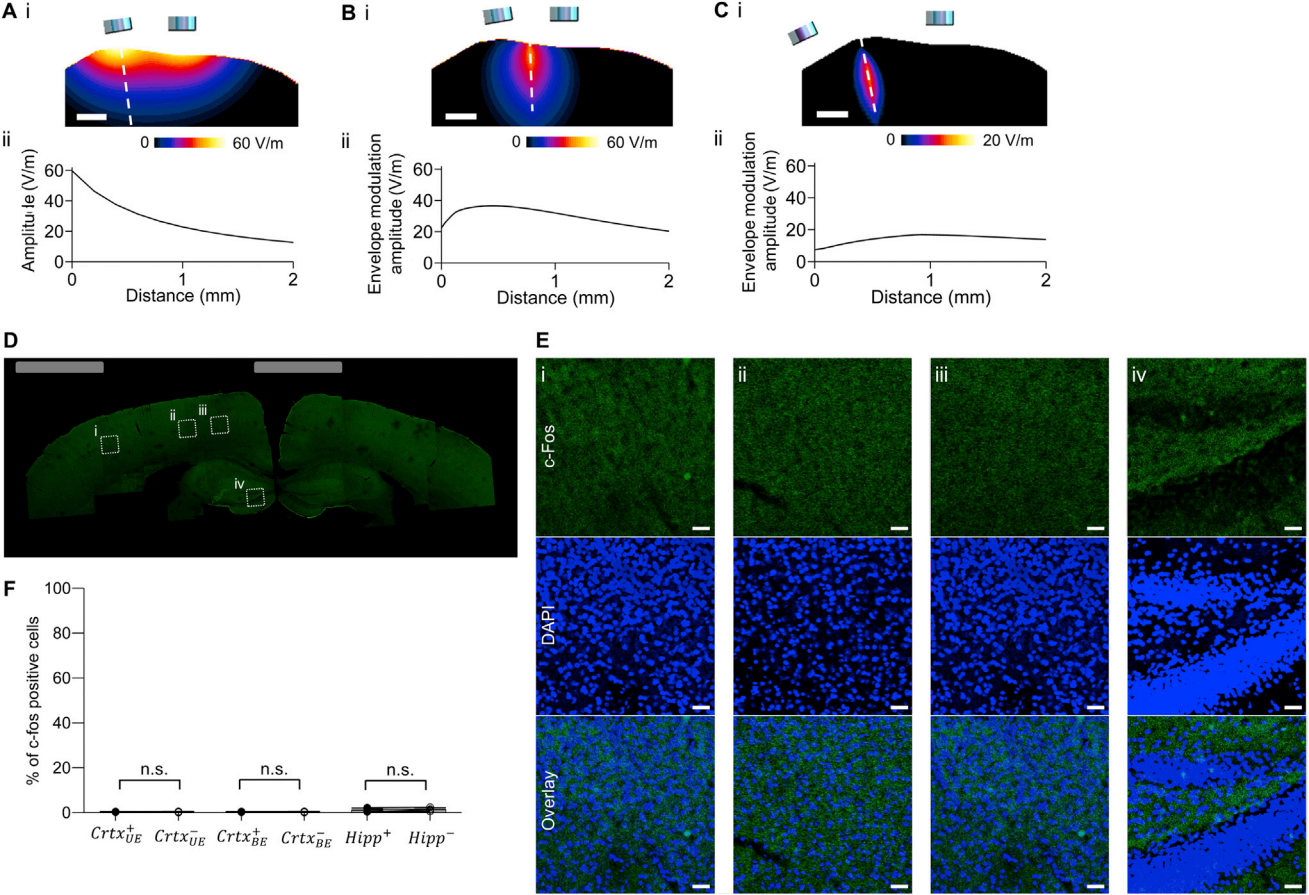
Application of TI to Stimulation of Mouse Hippocampus, Related to Figure 3 (A) Quasi-electrostatic finite element method (FEM) mouse model simulation of 10 Hz and 2 kHz stimulations, corresponding to Figures 3A–3C and Figures 3D–3F, respectively. Showing (i) field amplitude map $\left|E_{\hat{r}}\left(x,y\right)\right|$ of simulated fields along the direction $\hat{r}$ orthogonal to the brain surface, and (ii) plot of field amplitude $\left|E_{\hat{r}}\left(r\right)\right|$ along dashed line in (i) that is perpendicular to the brain surface. In this case, two alternating currents at a frequency of 10 Hz and amplitude of 125 μA were simulated at electrode sites with a 1.5 mm gap. (B) As in (A) but for TI stimulation, corresponding to Figures 3G–3I, showing the envelope modulation amplitude $\left|E_{AM\hat{r}}\left(x,y\right)\right|$. (C) As in (B) but for TI stimulation with a larger inter-electrode spacing, corresponding to (D-F). Scale-bars for (A), (B), (C) 1 mm. Distances are measured from the surface of the brain. Color-maps are values in V/m. Mouse anatomical model (x, y, z) resolution was (42 μm, 42 μm, 700 μm) respectively. (D–F) Experimental probing of hippocampal activation with TI stimulation but with a large inter-electrode distance. TI stimulation with anesthetized mice as in Figures 3G–3I but electrodes were placed at a larger distance from each other on the skull (relative to bregma: at anteroposterior (AP) −2 mm, mediolateral (ML) −0.25 mm, and AP −2 mm, ML 4.25 mm). Currents were applied in a 10 s-on, 10 s-off pattern for 20 min. Shown is a representative image montage of a slice of stimulated brain showing c-*fos* expression (stained with anti-c-*fos*, shown in green). Grey rectangles illustrate electrode lateral positions. Boxed regions are highlighted in (E). (E) C-fos (green) overlaid with 4′,6-diamidino-2-phenylindole (DAPI) (blue) staining to highlight individual cell nuclei, from boxed regions i to iv in (D). (F) Percentage of c-*fos*–positive cells within a DAPI-labeled cortical area (500 μm x 500 μm) underneath the electrode $\left(Crtx_{UE}^{+}\right)$, a contralateral cortex area ($Crtx_{UE}^{-}$; 500 μm x 500 μm), a cortex area (1500 μm x 500 μm) between the stimulating electrodes $\left(Crtx_{BE}^{+}\right)$, an area in the contralateral cortex area ($Crtx_{BE}^{-}$; 1500 μm x 500 μm), a dentate gyrus area (500 μm x 500 μm) in the hippocampus of the stimulated hemisphere $\left(Hipp^{+}\right)$, and a dentate gyrus area of the hippocampus in the contralateral (non-stimulated) hemisphere ($Hipp^{-};\;$ 500 μm x 500 μm). Bars show mean values ± SD, n = 4 mice. Significance was analyzed by one-way ANOVA with Bonferroni post hoc test; for full statistics see Table S2. Scale bar for (D) 0.5 mm; scale bars for (E) 25 μm.

We next aimed to stimulate a deep structure (i.e., mouse hippocampus) while not recruiting overlying structures (i.e., the cortex). We performed simulations like those we did before, but now for the mouse brain, and predicted that 10 Hz transcranial stimulation applied to sites at the surface of the skull (Figure S4A) would broadly recruit neural activity throughout both superficial and deep structures. In contrast, performing TI stimulation with a 10 Hz difference frequency (Figure S4B) would, in our models, result in a peak of 10 Hz envelope modulation at a deep site, with lower envelope modulation amplitudes in more superficial structures. Of course, such models make assumptions about brain electrical parameters and geometry that may vary from brain to brain (Peratta and Peratta 2010), possess limited spatial resolution (our anatomical mouse model had x, y, and z resolutions of 42 μm, 42 μm, and 700 μm, respectively) and do not take into account differences in neural excitability across cell types and brain regions. Thus, to assess whether TI stimulation could indeed recruit activity in deep neural circuits without driving overlying ones, we compared 10 Hz versus TI stimulation in anesthetized mice, using the immediate early gene c-*fos* as a marker of neural activity, as has been used previously to gauge the focality of brain stimulation, since c-*fos* functions in the mouse cortex and hippocampus as an indicator of activated neurons (Chen et al., 2015, Dragunow and Robertson, 1987, Reijmers et al., 2007).

**Figure 3:**
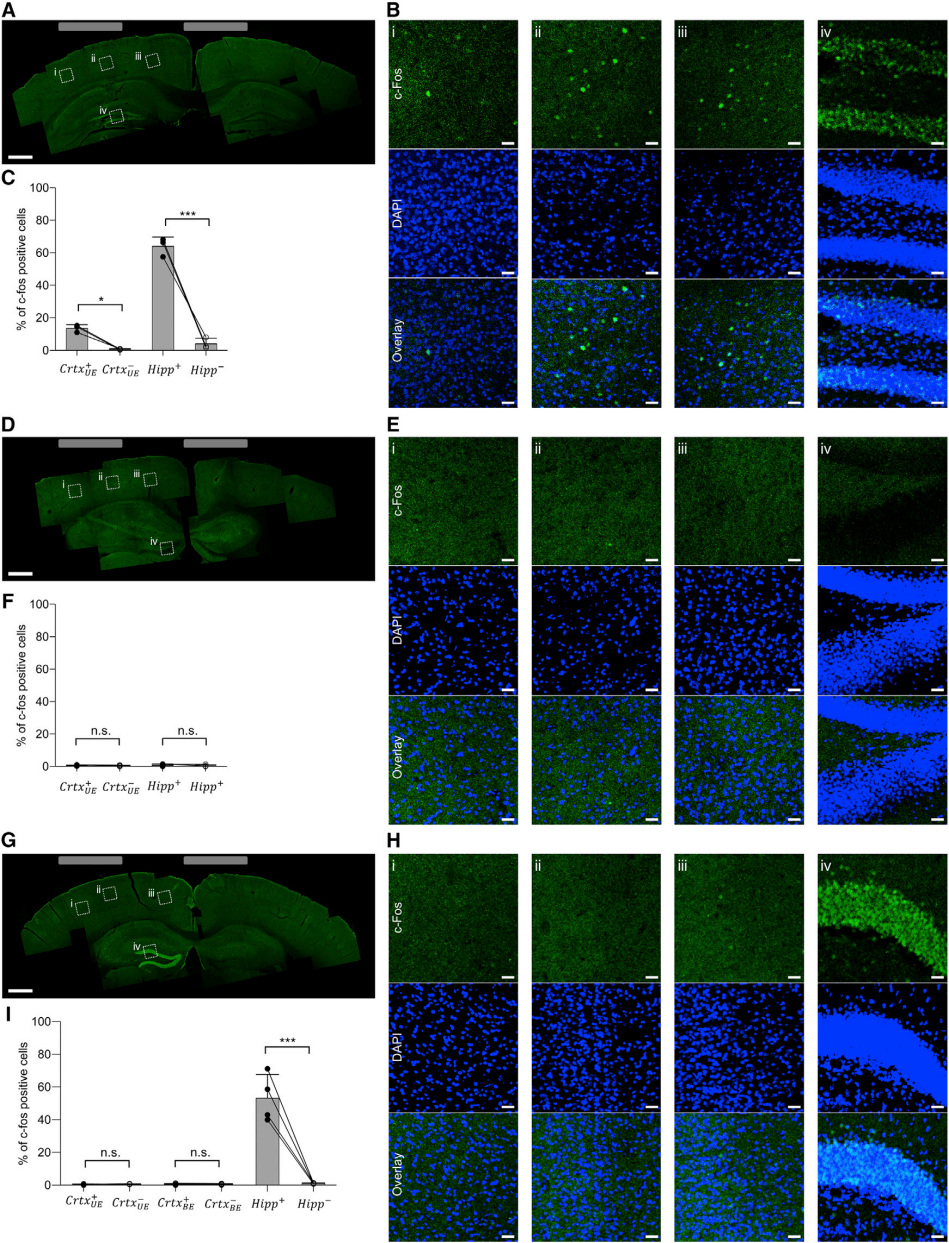
Application of TI to Stimulation of Mouse Hippocampus without Recruitment of Overlying Cortex (A) 10 Hz stimulation with anesthetized mice bearing two electrodes made of saline-filled tubes (1.5 mm outer diameter) placed on the skull surface (relative to bregma: at anteroposterior (AP) −2 mm, mediolateral (ML) −0.25 mm, and AP −2 mm, ML 2.75 mm). Currents (125 μA per electrode pair) were applied in a 10 s-on, 10 s-off pattern for 20 min. Shown is a representative image montage of a slice of stimulated brain showing c-*fos* expression (stained with anti-c-*fos*, green). Grey rectangles illustrate electrode mediolateral positions. Boxed regions are highlighted in (B). (B) c-fos (green) overlaid with 4′,6-diamidino-2-phenylindole (DAPI, blue) staining to highlight individual cell nuclei, from boxed regions i to iv from (A). (C) Percentage of c-*fos*–positive cells within a DAPI-labeled cortical area (500 μm x 500 μm) underneath the electrode $\left(Crtx_{UE}^{+}\right)$, a contralateral cortex area $(Crtx_{UE}^{-}$; 500 μm x 500 μm), a dentate gyrus area (500 μm x 500 μm) in the hippocampus of the stimulated hemisphere $\left(Hipp^{+}\right)$ and a dentate gyrus area of the hippocampus in the contralateral (non-stimulated) hemisphere ($Hipp^{-};\;$ 500 μm x 500 μm). Bars show mean values ± SD; n = 3 mice. (D–F) As in (A)–(C), but for the case where the currents are delivered at 2 kHz frequency; n = 4 mice in (F). (G–I) As in A-C, but for the case of TI stimulation with the lateral electrodes driven at 2 kHz and the medial electrodes driven at 2.01 kHz. The two pairs of electrodes were electrically isolated (see Figure S3 for description of isolation). In (I), c-*fos*–positive neurons were analyzed in the locations analyzed in (C) and (F), but also in a cortex area (1000 μm x 500 μm) between the stimulating electrodes $\left(Crtx_{BE}^{+}\right)$ and in the contralateral cortex area ($Crtx_{BE}^{-}$; 1000 μm x 500 μm); n = 4 mice. Significance in (C), (F), and (I) was analyzed by one-way ANOVA with Bonferroni post hoc test, ^∗^p < 0.05, ^∗∗∗^p < 0.00001; for full statistics for Figure 3, see Table S2; scale bars for (A), (D), and (G) represent 0.5 mm; scale bars for (B), (E), and (H) represent 25 μm.

As expected, 10 Hz transcranial stimulation (10 s on then 10 s off, for 20 min) resulted in widespread c-*fos* expression (measured 90 min after stimulation) in both the cortex and in the hippocampus underlying the electrodes (Figures 3A and 3B), with 13.6% ± 2.2% (mean ± SD used throughout) of cortical cells (as indicated by DAPI-stained nuclei) and 63.9% ± 5.7% of hippocampal cells c-*fos*-positive underneath the electrodes (Figure 3C). In contrast, there was essentially no c-*fos* activation on the contralateral side (see Table S2 for complete statistics for Figure 3). Driving the brain with a 2 kHz transcranial current (with the same current magnitude and durations as in the 10 Hz case) resulted in essentially no c-*fos* positive cells (Figures 3D–3F), in either the cortex or the hippocampus, and on either the electrode-bearing or contralateral side. In contrast, when TI stimulation was applied with frequencies of 2 kHz and 2.01 kHz (with the same current magnitude and duration as in the 10 Hz case), the hippocampus was strongly activated, Figures 3G and 3H, with c-*fos* in 53.12% ± 14.5% of DAPI-labeled cells (Figure 3I), not significantly different from that recruited by the 10 Hz stimulation (Figure 3C). But, despite the strong hippocampal recruitment, there was essentially zero c-*fos* in cortical cells—both at a site between the stimulating electrodes where the cortical envelope modulation field would be anticipated to be at its highest value in the cortex, with c-*fos* in 0.48% ± 0.47% of DAPI-labeled cells, and directly underneath an electrode, with c-*fos* in 0.32% ± 0.29% of DAPI-labeled cells (Figure 3I). Thus, TI stimulation can recruit neural activation in a deep structure such as the hippocampus without recruiting the overlying cortex. As a control experiment, we separated the electrodes by a larger distance, which would be expected to reduce the envelope modulation field amplitude (Figure S4C), and obtained no activation in the cortex or hippocampus (Figures S4D–S4F).

We did not observe seizures during or after any of these stimulation paradigms (i.e., 10 Hz, 2 kHz, or TI stimulation). Furthermore, c-*fos* staining was always observed only on the ipsilateral side of stimulation, and not in other analyzed regions, including below the hippocampus or in the contralateral hippocampus, consistent with a local (as opposed to propagating) neural activity profile. The high c-*fos* expression observed in the dentate gyrus is consistent with c-*fos* expression patterns observed in rats after strong, unilateral electrical stimulation of the hippocampus via an implanted electrode in animals treated with carbamazepine, which prevents seizures and also prevents bilateral c-*fos* staining upon unilateral stimulation (Dragunow and Robertson, 1987).

### Safety Characterization of TI Stimulation

**Figure 4:**
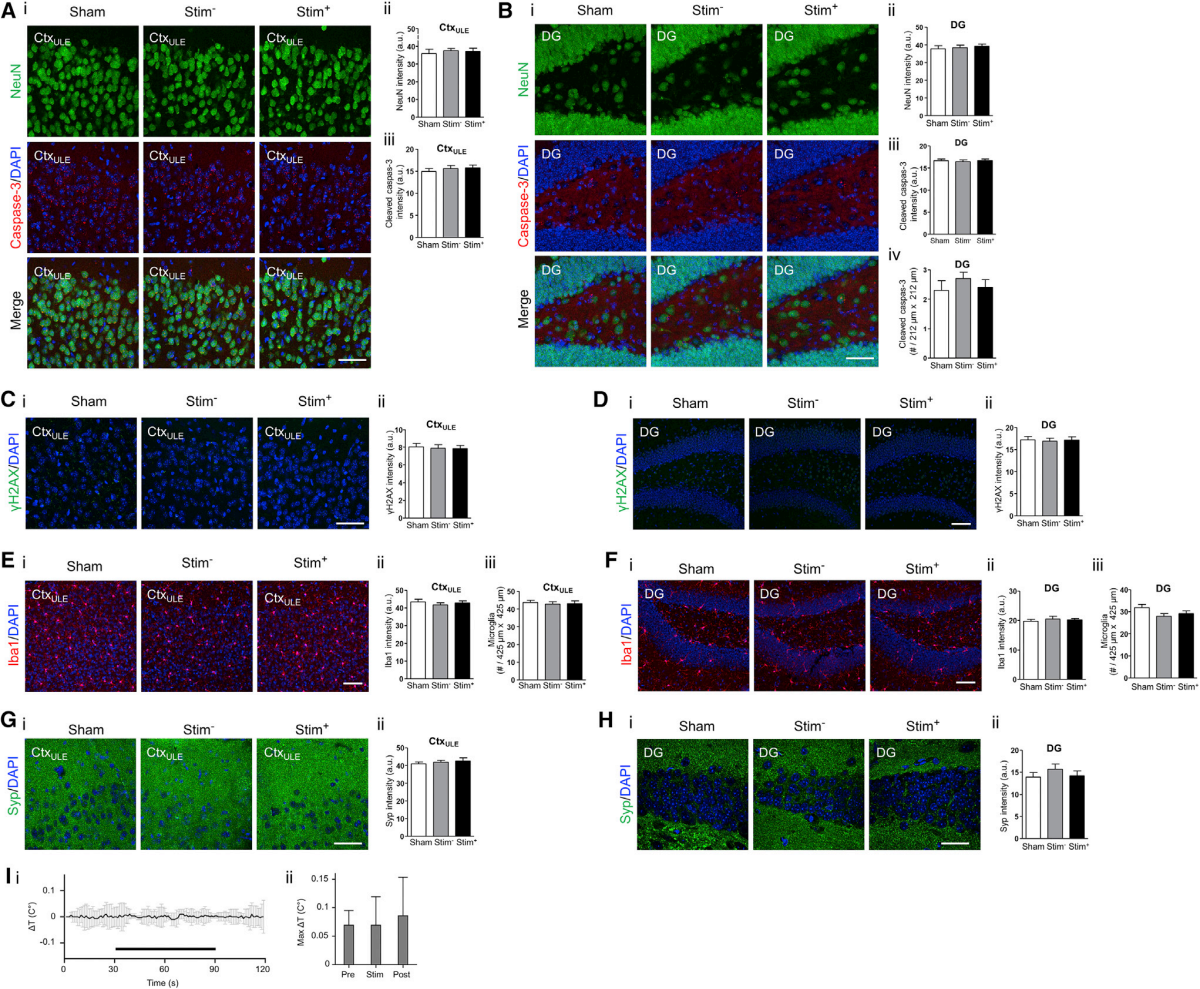
Safety Assessments for TI Stimulation (A–H) Immunohistochemical characterization of cellular and synaptic markers after TI stimulation of awake mice. Stimulating currents ($I_{1}$, 2.01 kHz, 125 μA; $I_{2}$, 2 kHz, 125 μA) were applied in a 10 s-on, 10 s-off pattern for 20 min with 0.5 s ramp-up and ramp-down periods, via two electrodes placed on the skull surface (relative to bregma: at anteroposterior (AP) −2 mm, mediolateral (ML) −0.25 mm, and AP −2 mm, ML 2.75 mm), as in Figure 3G–3I. For each panel, subpanels show (i) representative immunohistochemically stained slices and (ii and iii) mean ± SEM of immunohistochemical values as described below for individual panel caption sections. Stim^+^, brain regions from stimulated hemisphere; Stim^−^, brain regions from the contralateral, unstimulated hemisphere; Sham, brain regions from mice that underwent the same procedure but with $I_{1}$ and $I_{2}$ set to 0 μA. Significance was characterized using one-way ANOVA; n = 2 sections from 5 mice each. Scale bars for (i) are 50 μm. (A) NeuN staining and cleaved caspase-3 staining, from a cortical region underneath the lateral electrode (Ctx_ULE_). (ii) NeuN intensity. (iii) Cleaved caspase-3 intensity. (B) As in (A) but for the dentate gyrus of the hippocampus (DG), with additionally (iv) number of cleaved caspase-3 positive cells. (C) γH2AX staining from Ctx_ULE_ to assess DNA damage. (ii) γH2AX intensity. (D) As in (C) but from the DG. (E) Iba1 staining from Ctx_ULE_. (ii) Iba1 intensity. (iii) Number of Iba1-positive cells. (F) As in (E) but from the DG. (G) Synaptophysin (Syp) staining from Ctx_ULE_. (ii) Syp intensity. (H) As in (G) but from the DG. See Figures S5A–S5I for immunohistochemical assessment of cortex regions underneath the electrode that was located centrally, as well as between the electrodes; see Figures S5J–S5O for immunohistochemical assessment of CA1 region of the hippocampus. See Table S3 for full statistics of cortical and hippocampal regions. (I) Measurement of tissue temperature. High-frequency stimulating currents ($I_{1}$, 2 kHz, 500 μA; $I_{2}$, 2 kHz, 500 μA) were simultaneously applied with 0.5 ramp-up and ramp-down periods via two electrodes placed on the skull surface as in (A)–(H). The temperature of the brain tissue underneath the lateral electrodes was measured using an invasive thermocouple probe during 60 s of stimulation (‘Stim’ period) as well as 30 s before (‘Pre’ period) and 30 s after (‘Post’ period) stimulation. Plotted is (i) instantaneous change in brain temperature from baseline as a function of time; black bar indicates period of stimulation. (ii) Maximal increase in brain temperature from the baseline (i.e., pre-stimulation) mean temperature. Shown are mean ± SD; significance calculated via one-way ANOVA; p = 0.8091; n = 6 mice; see Table S3 for full statistics.

**Figure S5:**
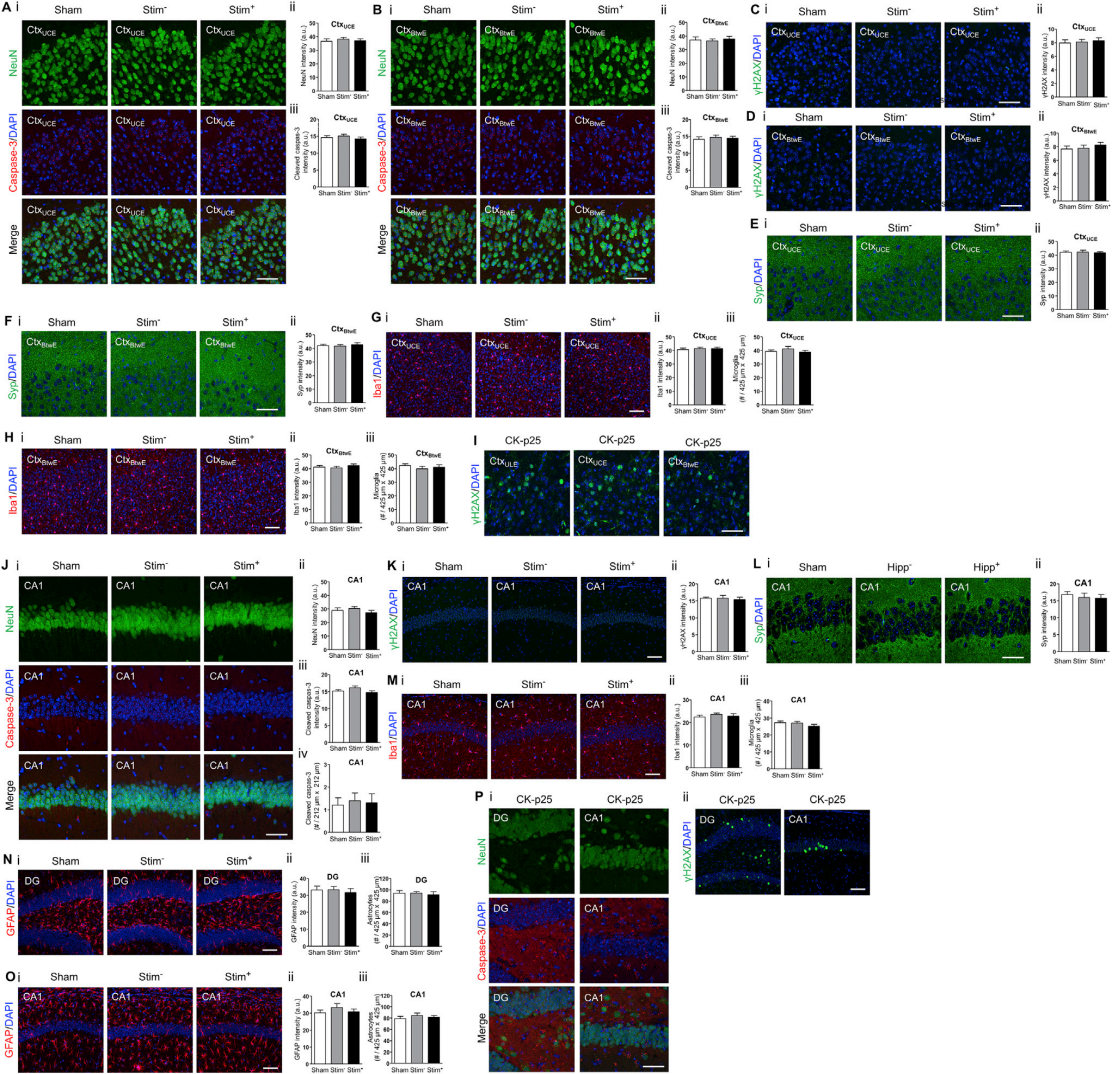
Safety Assessment of Temporal Interference Stimulation, Related to Figure 4 Immunohistochemical measurement of cellular and synaptic markers after TI stimulation (as in Figure 4) of awake mice showing (i) representative immunohistochemically stained slices and (ii-iii) mean ± s.e.m of immunohistochemical values as described below in the individual panel caption sections; Stim^+^, brain regions from stimulated hemisphere; Stim^−^, brain regions from the contralateral hemisphere that was not stimulated; Sham, brain regions from mice that underwent the same procedure but with current amplitudes of currents $I_{1}$ and $I_{2}$ set to 0 μA. Significance was characterized using one way ANOVA; n = 5 mice, 2 sections from each mouse; scale bars for (i) 50 μm. (A–I) Cortex. (A) NeuN staining and cleaved caspase-3 staining for a cortical region underneath the midline (central) electrode (Ctx_UCE_). (ii) NeuN intensity. (iii) Cleaved caspase-3 intensity. (B) As in (A) but for a cortical region between the electrodes (Ctx_BtwE_). (C) γH2AX staining for Ctx_UCE._ (ii) γH2AX intensity. (D) As in (C) but for Ctx_BtwE_. (E) Synaptophysin (Syp) staining for Ctx_UCE._ (ii) Syp intensity. (F) As in (E) but for Ctx_BtwE_. (G) Iba1 staining for Ctx_UCE_. (ii) Iba1 intensity. (iii) Number of Iba1 positive cells. (H) As in (G) but for Ctx_BtwE_. (I) γH2AX staining for cortical regions of CK-p25 mouse, an established mouse model of neurodegeneration, with neuronal atrophy, reduced synaptic density and pronounced DNA damage (Cruz et al., 2003, Dobbin et al., 2013, Kim et al., 2008), plotted here as a positive staining control for the utilized antibodies. (J–P) Hippocampus. (J) NeuN and cleaved caspase-3 staining for CA1 region of the hippocampus (CA1). (ii) NeuN intensity. (iii) Cleaved caspase-3 intensity. (iv) Number of cleaved caspase-3 cells. (K) γH2AX staining for CA1. (ii) γH2AX intensity. (L) Synaptophysin (Syp) staining for CA1. (ii) Syp intensity. (M) Iba1 staining for CA1. (ii) Iba1 intensity. (iii) Number of Iba1 positive cells. (N) GFAP staining for dentate gyrus of the hippocampus (DG). (ii) GFAP intensity. (iii) Number of GFAP-positive cells. (O) As in (N) but for CA1. (P) Staining for DG and CA1 regions of CK-p25 mouse, an established mouse model of neurodegeneration, with neuronal atrophy, reduced synaptic density and pronounced DNA damage (Cruz et al., 2003, Dobbin et al., 2013, Kim et al., 2008), plotted here as a positive staining control for the utilized antibodies. (i) NeuN and cleaved caspase-3 staining. (ii) γH2AX staining. See Table S3 for full statistics for this figure.

To characterize the safety profile of TI stimulation, we immunohistochemically examined cellular and synaptic molecular profiles in the cortex and the hippocampus after unilateral TI stimulation (2 kHz and 2.01 kHz, 10 s on then 10 s off, for 20 min), as in Figures 3G–3I, but in awake, behaving mice. Mice were sacrificed and transcardially perfused after a 24 hr recovery period to allow for detection of persistent effects (e.g., caspase-3 activation) after a bout of stimulation. Brain sections were fluorescently stained with antibodies for the neuronal marker NeuN (Wolf et al., 1996), the apoptotic marker cleaved caspase-3 (D’Amelio et al., 2012), the DNA damage marker γH2AX (Mah et al., 2010), the microglial marker Iba1 (Ito et al., 1998), the astrocyte marker GFAP (Eng et al., 2000), and the synaptic protein synaptophysin (Syp) (Tarsa and Goda 2002). We compared fluorescence profiles in the brain regions that were stimulated (Stim+) with fluorescence profiles in the contralateral, non-stimulated hemisphere (Stim−), as well as with fluorescence profiles in mice that underwent the same procedure but with current amplitudes set to 0 μA (Sham). We found that TI stimulation did not alter the neuronal density or affect the number of apoptotic cells (Figures 4A and 4B, Figures S5A and S5B, Figure S5J) or induce DNA damage (Figures 4C and 4D, Figures S5C and S5D, Figure S5K), at least as reflected by the stains above, relative to unstimulated or Sham stimulated brains. In addition, TI stimulation did not alter the intensity and density of Iba1 positive cells (Figures 4E and 4F, Figures S5G and S5H, Figure S5M) or GFAP-positive cells (Figures S5N and S5O), suggesting a lack of reactive microglia and astrocytes, respectively, in response to TI stimulation. Finally, TI stimulation did not alter synaptophysin intensity, suggesting no changes to synapse density (Figures 4G and 4H, Figures S5E and S5F, Figure S5L). See Table S3 for full statistics for these experiments, analyzed over cortical and hippocampal regions.

To assess whether high-frequency electric fields heat the brain, we measured brain temperature during stimulation with 2 kHz fields (60 s with 0.5 s ramp-up and ramp-down periods) that were applied via an electrode configuration as in Figures 4A–4H. We measured temperature with a 1-mm-diameter thermocouple probe inserted into the cortex underneath the lateral electrode. We found that the maximal temperature increase at this cortical location during stimulation was 0.069 ± 0.05 C° (mean ± SD increase from baseline temperature; n = 6 mice). This change in brain temperature was not larger than the largest spontaneous deviations from baseline seen during the pre- and post-stimulation periods (Figure 4I; p = 0.81, one-way ANOVA; see Table S3 for full statistics for Figure 4I).

### Steerable Probing of Motor Functionality without Electrode Movement

**Figure 5:**
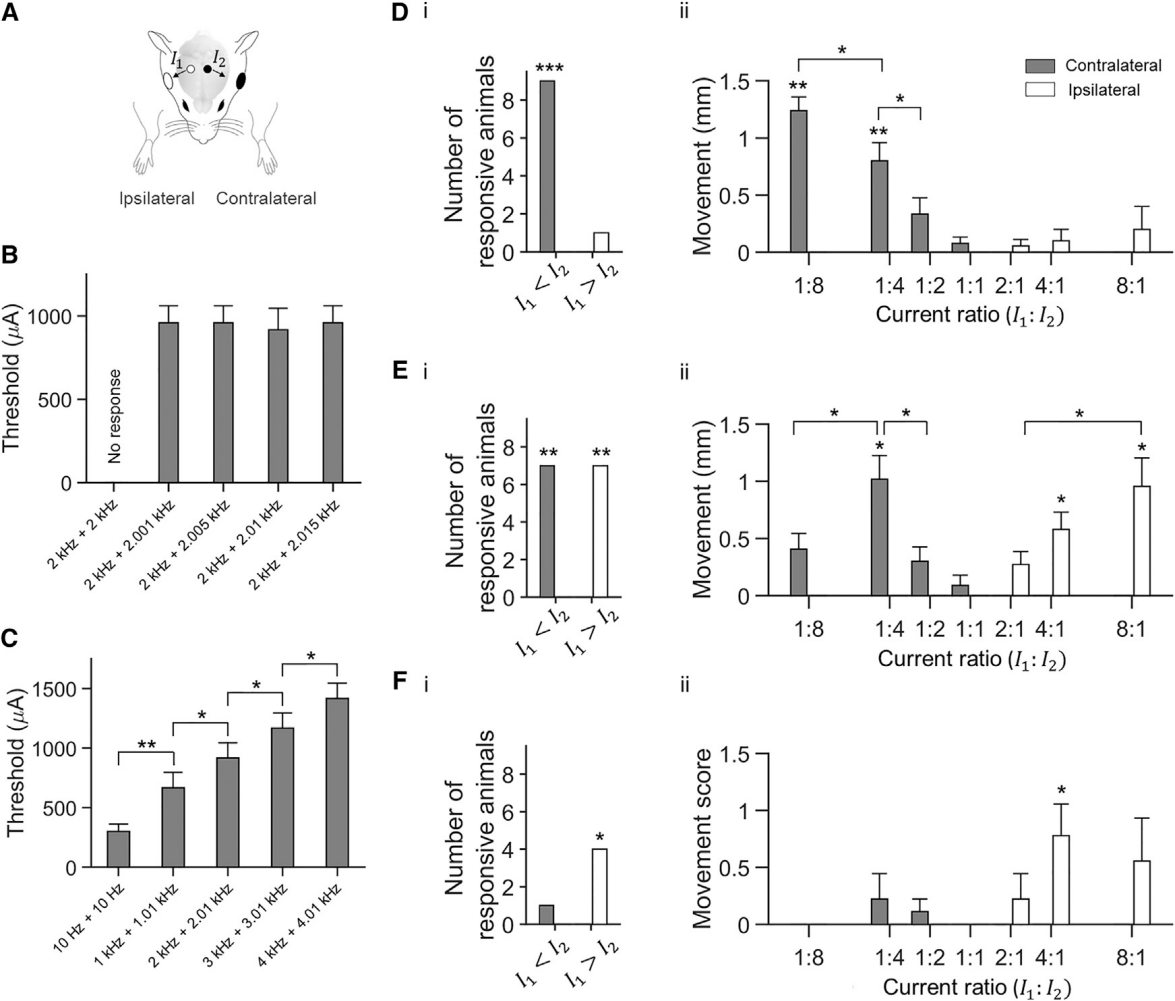
Application of TI to Steerable Probing of Mouse Motor Cortex Functionality (A) Currents $I_{1}$ and $I_{2}$ were applied simultaneously (0.5 s ramp-up, 6 s stimulation, 0.5 s ramp-down) to anesthetized head-fixed mice and motor activity was video-recorded (including 1.5 s pre-stimulation and post-stimulation periods). Current $I_{1}$ was applied via a 1 mm diameter skull electrode (white circle; relative to bregma, AP −1.5 mm, ML +2 mm, n = 5 mice; or AP −1.5 mm, ML −2 mm, n = 4 mice) paired with a 5-8 mm diameter electrode (white ellipse). Current $I_{2}$ was applied via a similarly sized skull electrode (black circle; relative to bregma, AP −1.5 mm, ML −0.5 mm, n = 5 mice; or AP −1.5 mm, ML +0.5 mm, n = 4 mice) paired with a 5-8 mm diameter electrode (black ellipse). (B and C) Characterization of motor threshold. Current ratio $I_{1}\text{:}I_{2}$ was fixed at 1:4. Shown is mean motor threshold ± SD (n = 6 mice). Significance calculated using one-way ANOVA followed by post-hoc test with Bonferroni correction for multiple comparisons. (B) Comparison of motor thresholds with TI stimulation at different difference frequencies and a fixed 2 kHz carrier frequency; p = 0.88; see Table S4 for full statistics for (B). (C) Comparison of motor thresholds with TI stimulation at different carrier frequencies and fixed 10 Hz difference frequency; ^∗^p < 0.05, ^∗∗^p < 0.0005; see Table S4 for full statistics for (C). (D–F) Steerable motor cortex activation. Current $I_{1}$ at a frequency of 1 kHz and current $I_{2}$ at a frequency of 1.01 kHz were applied at different amplitude ratios $I_{1}:I_{2}$ but with a fixed current sum $I_{1}+I_{2}$ (776 μA ± 167 μA; mean ± SD; n = 9 mice). (D) Evoked movements of the forepaws. (E) Evoked movements of the whiskers. (F) Evoked movements of the ears. (i) Number of animals, out of a total of 9 animals, in which the TI stimulation with $I_{1}:I_{2}$ current ratios of 1:2, 1:4 or 1:8 (‘$I_{1}<I_{2}$’), and with $I_{1}\text{:}I_{2}$ current ratios of 2:1, 4:1 or 8:1 (‘$I_{1}>I_{2}$’) evoked a movement ipsilateral to $I_{1}$ electrode (white) or contralateral to $I_{1}$ electrode (gray). Significance of number of responders was characterized using Fisher’s exact test; ^∗^p < 0.05, ^∗∗^p < 0.005, ^∗∗∗^p < 0.00001. See Table S4 for full statistics. (ii) Evoked movements ipsilateral to $I_{1}$ electrode (white) and contralateral to $I_{1}$ electrode (gray) at different current ratios $I_{1}:I_{2}$. Shown values are mean ± SEM; n = 9 mice. Ear movements were visually scored on the following scale: 0, no movement; 1, weak movement; 2, strong movement; 3, very strong movement. Significance of evoked movement for each current ratio was characterized using an unpaired t test versus null hypothesis of no movement, thresholding at p < 0.0025, Bonferroni corrected for multiple comparisons; ^∗^p < 0.0025, ^∗∗^p < 0.00001; significance between current ratios was calculated using one-way ANOVA followed by post hoc test with Bonferroni correction for multiple comparisons; ^∗^p < 0.05. See Table S4 for full statistics.

We next explored the capability of TI stimulation to activate neurons so as to drive movements. Using ketamine-xylazine anesthetized mice, we applied a current $I_{1}$ via an electrode that was positioned on the skull above the motor cortex region associated with a movement of the contralateral forepaw and a current $I_{2}$ via a second electrode that was positioned on the contralateral skull, above the motor cortex region associated with movement of the whiskers ipsilateral to the $I_{1}$ electrode (Figure 5A) (Tennant et al., 2011).

We first established the motor threshold by systematically increasing the current sum $I_{1}+I_{2}$ in steps of 50 μA while keeping the current ratio $I_{1}:I_{2}$ fixed at 1:4. We found that if $I_{1}$ and $I_{2}$ were applied with a carrier frequency of 2 kHz and a difference frequency of 10 Hz, the stimulation evoked a 10 Hz periodic movement of the contralateral forepaw with a motor threshold of 916 ± 129 μA (Figure 5B; mean ± SD, n = 6 mice). If $I_{1}$ and $I_{2}$ were applied at the same frequency, no motor movement was observed (n = 9 mice; assessed up to a current sum value of 2 mA). Changing the difference frequency between 1 Hz and 15 Hz changed the motion frequency accordingly, but not the motor threshold (p = 0.88; one-way ANOVA; n = 6 mice; Figure 5B and Movie S1; see Table S4 for full statistics associated with Figure 5B). Increasing the carrier frequency from 1 kHz to 4 kHz linearly increased the motor threshold with a slope of 250 μA/kHz (linear regression, $R^{2}=0.99$; Figure 5C and Movie S2; see Table S4 for full statistics associated with Figure 5C); a 5 kHz carrier resulted in no response at the maximum current sum value tested (2 mA).

We next sought to test whether steering the site of stimulation by changing the current ratio $I_{1}:I_{2}$, as in our physics experiments (Figures 2C–2E), would shift the site of motor cortex activation. We changed the current ratio $I_{1}:I_{2}$, keeping $I_{1}+I_{2}$ fixed, and measured movements evoked in the forepaws, whiskers, and ears (see Movie S3 for representative video). We found that when $I_{1}<I_{2}$ (that is, stimulation was steered toward the $I_{1}$ electrode), TI stimulation evoked a movement of the forepaw (Figure 5Di, p = 0.00004, Fisher’s exact test, n = 9 mice) and the whiskers (Figure 5Ei, p = 0.002, n = 9 mice) contralateral to the $I_{1}$ electrode, but no movements ipsilateral to the $I_{1}$ electrode (Figures 5Di–5Fi; see Table S4 for full statistics associated with Figures 5Di–5Fi). In contrast, when $I_{1}>I_{2}$ (stimulation was steered toward the $I_{2}$ electrode), TI stimulation evoked a movement of the whiskers (Figure 5Ei, p = 0.002, n = 9 mice) and ear (Figure 5Fi, p = 0.03, n = 9 mice) ipsilateral to the $I_{1}$ electrode but no movements contralateral to the $I_{1}$ electrode (Figures 5Di–5Fi).

The movement of the forepaw contralateral to the $I_{1}$ electrode was maximal when the current ratio $I_{1}:I_{2}$ was 1:8, i.e., our condition in which stimulation was maximally steered toward the $I_{1}$ electrode (1.24 ± 0.36 mm, mean movement ± SD used throughout; p = 0.000007, unpaired t test versus null hypothesis of no movement, thresholding at p < 0.0025 Bonferroni corrected for multiple comparisons; n = 9 mice) and gradually decreased as the current ratio increased (Figure 5Dii). (Perhaps because the electrodes were not placed symmetrically, the movement of the forepaw ipsilateral to the $I_{1}$ electrode, though not statistically significant, showed an opposite pattern, with a maximal movement when the current ratio was 8:1, i.e., when stimulation was maximally steered toward the $I_{2}$ electrode [Figure 5Dii, 0.2 ± 0.6 mm, p = 0.35, n = 9 mice].) The movement of the whiskers contralateral to the $I_{1}$ electrode was maximal when the current ratio $I_{1}:I_{2}$ was 1:4, i.e., stimulation was partially steered toward the $I_{1}$ electrode (0.4 ± 0.41 mm, p = 0.018, n = 9 mice) and gradually decreased as the current ratio varied from this maximum (Figure 5Eii; the 1:8 condition steers the stimulation more laterally than the 1:4 condition, which perhaps stimulated cortical regions more lateral to the whisker region [Tennant et al., 2011], and thus elicited a lower whisker movement amplitude than that elicited by the 1:4 condition). In contrast, perhaps due to the asymmetrical location of our electrodes, the movement of the whisker ipsilateral to the $I_{1}$ electrode was maximal when the current ratio $I_{1}:I_{2}$ was 8:1, i.e., stimulation was maximally steered toward the $I_{2}$ electrode (0.96 ± 0.75 mm, p = 0.0016, n = 9 mice) and gradually decreased as the current ratio decreased (Figure 5Eii). A similar trend was seen for the ears (Figure 5Fii; see Table S4 for full statistics associated with Figures 5Dii–5Fii). Thus, TI stimulation can support steering of brain stimulation without physical electrode movement, resulting in tunable elicitation of movements.

## Discussion

In this paper, we present TI stimulation, validating the concept by using modeling as well as both physics and neurophysiology experiments, and demonstrate its utility by performing stimulation of a deep region (mouse hippocampus) without stimulating overlying neurons (cortex), as well as steerable brain stimulation of motor patterns without physical electrode movement. Future studies, perhaps using larger numbers of electrodes and multiple sets of interfering fields, may be able to pinpoint even smaller regions of the brain, or multiple regions of the brain. An open question is how small a focal volume may be achieved. At some point, inhomogeneities in the gray and white matter of the brain may cause difficulty in improving the resolution below that spatial scale, although MRI scans and data-driven sculpting of the electric fields may be able to compensate for this to some degree.

How generalizable might be the effects observed here? There have been reports that strong kHz-frequency electric fields can block the propagation of compound action potentials in peripheral nerves (Cuellar et al., 2013, Kilgore and Bhadra, 2014). Such effects were localized to the immediate vicinity of the electrodes, leaving regions a few millimeters away, perhaps where the magnitude of the fields was lower, unaffected. The magnitude of the fields used in our study to transcranially recruit neural activity in the brain were perhaps one to two orders of magnitude weaker than in these earlier studies, so we anticipate that such effects may have been minimal in our study. This is consistent with our repeatable observation of a lack of physiological effect of 2 kHz electric fields on brain activity. However, future studies might seek to explore how stronger kHz-frequency electric fields affect the brain. Such data might also present an upper limit to the field strengths applicable for TI stimulation. There has been a report of kHz-frequency transcranial electric field stimulation (1–5 kHz) that resulted in neural plasticity similar to that resulting from anodal DC stimulation (Chaieb et al., 2011), but a later report found that 5 kHz transcranial electric fields, grouped in theta burst patterns, did not result in neural plasticity (Kunz et al., 2017). In our current dataset, we did not observe effects of kHz-frequency electric fields beyond a brief transient that was observed when short ramp-up times were used (but not with longer ramp-up times), both suggesting that, in studies using kHz-frequency fields, subtle parameters of the stimulation may help determine exactly what effects on neural activity result and presenting an area for future exploration. We found that TI stimulation at amplitudes sufficient to recruit deep brain structures, such as the hippocampus, did not alter the neuronal and synaptic integrity of the underlying tissue 24 hr after stimulation, at least as reflected by the stains we used. Additional time points other than 24 hr post stimulation may provide, in the future, a more detailed picture of the safety of TI stimulation. Furthermore, the safety profile of TI stimulation associated with evoked behavior patterns, such as those utilized here, should be explored in the future.

Given the remarkable therapeutic benefits of DBS for patients with otherwise treatment-resistant movement and affective disorders (Kringelbach et al., 2007), the prospects for noninvasive DBS using electricity are potentially exciting. Other methods for noninvasive DBS have been proposed, e.g., using transcranial ultrasound (Legon et al., 2014) or using expression of heat-sensitive receptors and injection of thermomagnetic nanoparticles (Chen et al., 2015), but the unknown mechanism of action (Plaksin et al., 2014) and the need to genetically manipulate the brain, respectively, may limit their immediate use in humans. TI stimulation may thus represent a practical strategy for noninvasively stimulating neurons deep in the brain. It uses familiar and well-tested electric fields (Stavroulakis, 2014, International Commission on Non-Ionizing Radiation Protection, 2010) and does not require chemical or genetic manipulation of the brain. We anticipate that it might rapidly be deployable into human clinical trials, as well as studies of the human brain.

## STAR★Methods

### Key Resources Table

**Table:** 

REAGENT or RESOURCE	SOURCE	IDENTIFIER
**Antibodies**		

c-Fos	Santa Cruz Biotechnology	CAT#:sc-52
NeuN	Synaptic Systems	CAT#:266004
GFAP	Cell Signaling Technology	CAT#:12389
Iba1	Wako Cehmicals	CAT#:019-19741
Synaptophysin, SVP-38	Sigma-Aldrich	CAT#:S5768
Cleaved Caspase-3 (Asp175)	Cell Signaling Technology	CAT#:9664
γH2AX (anti-phospho-histone H2A.X)	Millipore	CAT#:05-636
Alexa Fluor 488 secondary antibody	ThermoFisher Scientific	CAT#:A11008
Alexa Fluor 594 secondary antibody	ThermoFisher Scientific	CAT#:A11012
Alexa Fluor 647 secondary antibody	ThermoFisher Scientific	CAT#:A21244
DAPI	Sigma-Aldrich	CAT#:10236276001

**Chemicals, Peptides, and Recombinant Proteins**		

SignaGel conductive gel	ParkerLabs	CAT#:15-25
ProLong Gold anti-fade reagent	Invitrogen, ThermoFischer Scientific	CAT#:P36930
Dental cement	Parkell	CAT#:S380
Ten20 conductive paste	Weaver and Company	CAT#:10-20-4T

**Experimental Models: Organisms/Strains**		

Mouse: C57BL/6	Taconic Biosciences; Jackson laboratory	Cat#:C57BL/6NTac; 000664

**Software and Algorithms**		

MATLAB	MathWorks	RRID:SCR_001622
LabView	National Instruments	RRID:SCR_014325
pClamp	Molecular Devices	RRID:SCR_011323
Sim4Life	Zurich MedTech	https://www.zurichmedtech.com/sim4life/

**Other**		

Polyimide tubing	Vention Medical	CAT#:141-0092
2” X 2” Re-Usable TENS/EMS Unit Electrode Pads with Gel	Gurin Products	CAT#:TE110-2x2WC-10
Autopatcher	Kodandaramaiah, Suhasa B., et al. Nature protocols 11.4 (2016): 634-654.	N/A
Silver wire, diam. 0.25 mm	Sigma-Aldrich	CAT#:265578

### Contact for Reagent and Resource Sharing

Further information and requests for resources and reagents should be directed to and will be fulfilled by the Lead Contact, Ed Boyden (esb@media.mit.edu).

### Experimental Model and Subject Details

#### Mouse: C57BL/6

Sex: Male.
Age: 8–12 weeks old.
Source: All animals were purchased from Taconic Biosciences.
Housing and husbandry: Mice were housed in standard cages in the MIT animal facility with ad libitum food and water in a controlled light-dark cycle environment, with standard monitoring by veterinary staff.
Allocation of animals to experimental groups: Randomly assigned.
Committee approval: All animal procedures were approved by the Massachusetts Institute of Technology (MIT) Committee on Animal Care (CAC, Protocol Number: 1115-111-18), and all experiments conformed to the relevant regulatory standards.

#### Mouse: CK-p25

Sex: Male.
Age: 4 months old.
Source: The CK-p25 transgenic mouse was created in the Tsai lab (Cruz et al., 2003)
Housing and husbandry: Mice were housed in standard cages in the MIT animal facility with ad libitum food and water in a controlled light-dark cycle environment, with standard monitoring by veterinary staff.
Allocation of animals to experimental groups: Randomly assigned.
Committee approval: All animal procedures were approved by the Massachusetts Institute of Technology (MIT) Committee on Animal Care, and all experiments conformed to the relevant regulatory standards.

### Method Details

#### Design and Implementation of the TI Stimulator

The kHz currents were generated using a custom made device consisting of two electrically isolated current sources. To isolate the channels, each waveform was supplied via a balanced pair of current sources that were driven in precisely opposite phase with a ground electrode carrying any imbalance currents (< 1%) from the paired current sources, preventing charging of the body relative to earth ground (Figure S3A). Each current source reliably drove 2 mA of current on 1 kΩ loads up to a frequency of 10 kHz with a resolution of 0.02Hz (Figures S3C and S3D). At load resistances higher than 10 kΩ the current source eventually saturated. The current output had a leakage level < 0.1 μA root mean square (RMS) at 100 kHz bandwidth, measured on a 1 kΩ load resistor with a differential amplifier (7A22, Tektronix). The total harmonic distortion of the current source was < 0.08% at 100 Hz and < 0.4% at 10 kHz (measured with 9 harmonics on a 1 kΩ load resistor). The total harmonic distortion and frequency cross-talk were measured using an FFT spectrum analyzer (SR770, Stanford Research). When the two current sources were applied to a common conductive load, e.g., a resistive bridge (Figure S3B) or a saline bath (Figure S3E), the cross-talk at the terminals of each channel was < 0.1%, allowing almost 100% of the interference to build up inside the load. In comparison, without the anti-phasic drive, approximately 30% cross-talk was measured at both the channel terminals and inside the conductive medium (Figure S3B, Figure S3F).

#### In-Vivo Rodent Electrophysiology

##### Surgical Procedures

On the day of the experiment, the mice were injected with Meloxicam (1mg/kg) and buprenorphine (0.1mg/kg) and anesthetized with 1%–2% (vol/vol) isoflurane in oxygen. Ophthalmic ointment (Puralube Vet Ointment, Dechra) was applied to the eyes. The scalp and the ventral torso were shaved and sterilized with Betadine and 70% ethanol. Two electrodes made of saline-filled polyimide tubes (Vention Medical Inc) with 1.5 mm outer diameter and 1.4 mm inner diameter or two electrodes made of adhesive electrogel with 1.5 mm diameter (SignaGel, ParkerLabs) were affixed to the skull (polyimide tubes were affixed using dental acrylic). During cortical recording, the positions of the skull electrodes relative to bregma were anteroposterior (AP) −1 mm, mediolateral (ML) −1.5 mm, and AP −1 mm, ML −2 mm; during hippocampus recording, their positions relative to bregma were: AP −2 mm, ML 0.25 mm, and AP −2 mm, ML −2.75 mm.

##### In Vivo Transcranial Stimulation

Transcranial stimulation was applied to anesthetized mice via the two skull electrodes, described above. Each skull electrode was paired with a cloth-base electrode (11 mm diameter conductive area; EL504, BioPac Inc) that was attached to the ventral torso with adhesive electrode gel (for the experiments of Figure 1**D-F,Ii**; SignaGel, Parker Laboratories Inc; 10-15 mm spacing between the edge of the conductive area of the torso electrodes) or with an adhesive electrode gel based electrode (SignaGel, ParkerLabs) on the cheeks (for the experiments of Figure 1**G-H,Iii**; approximately 11 mm diameter conductive area). Stimulation was applied for 1.5-2 s periods, with 0.25-0.5 s duration ramp-on periods and 0-0.5 s duration ramp-off periods, with at least a 2 s rest period between consecutive stimulations.

##### Whole-Cell Patch Clamp Recording

In vivo whole cell patching in current clamp mode (i.e., 0 pA holding current) was conducted in the cortex (depth of ∼500 μm below the dura) and CA1 layer of the hippocampus (depth of 1131 ± 157 μm below the dura) of anesthetized mice with an autopatcher (Kodandaramaiah et al., 2012). Data were acquired using pClamp software (Molecular Devices) at a 400 kHz sampling rate. Patch electrodes were pulled from thin-walled borosilicate glass capillary tubing using a P-97 puller (Sutter Instruments). Tip electrode resistance was 4.6–7.4 MΩ in artificial cerebrospinal fluid (ACSF), containing 126 mM NaCl, 3 mM KCl, 1.25 mM NaH2PO4, 2 mM CaCl2, 2 mM MgSO4, 24 mM NaHCO3 and 10 mM glucose). The patch electrode solution consisted of (in mM) potassium gluconate 122.5, KCl 12.5, KOH-HEPES 10, KOH-EGTA 0.2, Mg-ATP 2, Na3-GTP 0.3, NaCl 8 (pH 7.35, mOsm 296), with 0.2–0.4 mg/ml biocytin added immediately before use. Capacitance, series resistance and input resistance were frequently measured throughout recording to monitor patch quality and cell health, using 10-pA hyperpolarization/depolarization square current pulses; a 300 pA ramp depolarization over 500 ms was used for AP generation.

##### Data Analysis

Data were analyzed using MATLAB (MathWorks). The recorded traces from each neuron were split into blocks corresponding to each trial within an experiment. Each block consisted of a single stimulation period of 1.5 −2 s duration with 1 s of baseline recorded before and after each stimulation period. To reduce stimulation artifacts for spike identification, traces were filtered using a 5^th^ order Butterworth band-stop filter with cutoff frequencies of 1 kHz and 15 kHz and then with a 3^rd^ order Butterworth high-pass filter with a cutoff frequency of 100 Hz (representative traces from the cortex are shown in the Supplemental Materials without filtering and after filtering with only the band-stop filter; representative traces from the hippocampus are shown after filtering with only the band-stop filter). Single spikes were identified using a running window average that picked out depolarizations of > 40 mV above baseline, which were “peaky” (that is, exhibited amplitudes larger than the average amplitudes of the nearest 3 data points before and after, by > 0.001 mV). Consecutive spikes with inter-spike interval ≤ 15 ms, which occurred during a period of 50 ms or less, were defined as a spiking burst. Mean spiking frequency during stimulation periods (not including the ramping periods) was computed for each stimulation block and then averaged across neurons for each stimulation condition. Mean spontaneous firing rate was computed by a similar averaging of the firing rates across neurons, but for the 1 s interval before stimulation began. In the case of control 1 kHz or 2 kHz stimulation with no TI, we analyzed data from all complete blocks. Mean membrane potential was computed for a 500 ms period before the onset of 2 kHz or 1 kHz stimulation and was compared with a similar 500 ms period 1 s after stimulation onset, by dividing each period to 10 equally sized epochs and averaging across epochs. Overall, 18 neurons from 8 mice were analyzed with a minimal and maximal number of neurons per mouse of 1 and 4, respectively.

#### In-Vivo Stimulation Followed by c-*fos* Staining

##### Surgical Procedure

On the day of the experiment, to reduce background fos staining, mice were kept undisturbed in the dark, with full access to food and water, for at least an hour after being transferred from the animal facility to the experimental procedure room. Mice were injected with Meloxicam (1mg/kg) and buprenorphine (0.1mg/kg) and anesthetized with 1%–2% (vol/vol) isoflurane in oxygen. The scalp and the ventral torso were shaved and sterilized with Betadine and 70% ethanol. Two cloth-base electrodes with 11 mm diameter conductive area (EL504, BioPac) were attached to the ventral torso with saline electrode gel (SignaGel, Parker Laboratories). The spacing between the edge of the two conductive areas was 10-15 mm. The mice were then placed in a custom stereotax, with ophthalmic ointment (Puralube Vet Ointment, Dechra) applied to the eyes, and with Betadine and 70% ethanol used to sterilize the surgical area. Two polyimide tubes (Vention Medical) with 1.5 mm outer diameter and 1.4 mm inner diameter were affixed to the skull using dental acrylic (C&B Metabond, Parkell). One polyimide tube was located at stereotactic coordinates (relative to bregma) of anteroposterior −2 mm, mediolateral, −0.25 mm. The second polyimide tube was located to the left of the first tube, with a gap between the edges of the electrodes of between 1.5 and 4 mm. Once the dental acrylic set (∼20 min), the mice were removed from the stereotactic apparatus and placed in a custom-built low profile holder. The polyimide tubes were filled with saline solution and a silver wire of 0.25 mm diameter (Sigma-Aldrich) was inserted into each tube for connection to the stimulator.

##### In Vivo Transcranial Stimulation

The cranial tube electrodes and the cloth-base ventral torso electrodes were connected to the stimulator so that each cranial tube electrode was paired with one cloth-base ventral torso electrode. The complex impedance between each pair of electrodes was established by applying short currents of low amplitude (10 Hz and 2 kHz, 10 μA, 0.5 s) and measuring the applied current and resultant voltage waveforms. When the resistance between a pair of electrodes was higher than 1 MΩ, a dental drill was used to thin the skull area inside polyimide tubes (high impedance often resulted from a layer of dental acrylic that leaked into the polyimide tubes and hardened on the skull in that area). 10 s intervals of electrical stimulation, with 0.25 s ramp-on and ramp-off periods, were applied interspersed with 10 s rest intervals over a 20 min experimental time course. Mice were sacrificed after 90 min to allow for c-*fos* expression.

##### Histology, Immunohistochemistry, and Imaging

Mice were deeply anesthetized with ketamine/xylazine and sacrificed, then transcardially perfused with cold phosphate-buffered saline (PBS) followed by cold 4% paraformaldehyde (PFA) in 1 x PBS. The brains were dissected and post-fixed in 4% PFA in PBS at 4°C overnight. Free-floating vibratome coronal sections (35 μm) were cut and incubated in a blocking solution containing 10% normal donkey serum, 0.2% Triton X-100, 3% bovine serum albumin, and 0.02% sodium azide in 1 x PBS for 2 hr at room temperature (RT). Sections were labeled with primary anti-c-Fos antibody (1:400; sc-52; Santa Cruz Biotechnology, USA) in the blocking solution at 4°C overnight, followed by the Alexa488-conjugated (1:1000; Invitrogen, ThermoFisher Scientific) secondary antibody for 1 hr at RT. Slices were incubated for 20min in 1 x DAPI dye (Invitrogen, ThermoFisher Scientific) in PBS at RT to label cell nuclei. Samples were then washed 4 × 15 min in PBS with 0.1% Triton X-100. Immunolabeled brain sections were mounted onto glass slides using ProLong Gold anti-fade reagent (Invitrogen, ThermoFisher Scientific, USA) and stored at −20°C. Images of the cortical and hippocampal areas from the stimulated and unstimulated sides of the brain were acquired using a high-resolution multi-channel (sequential) scanning confocal microscope (LSM 510, Zeiss, Germany), using a 10x air objective (NA 0.45).

##### c-*fos* Quantification

Greyscale images were analyzed using MATLAB (MathWorks). Each greyscale image was processed with contrast-limited adaptive histogram equalization (128 tiles per image) followed by Wiener adaptive noise-removal lowpass filtering (using 5x5 neighboring pixels to estimate the local image mean and SD). DAPI and GFP greyscale images were converted to black and white (BW) masks with global image thresholds established using Otsu’s method. BW masks were smoothed using a morphological disc kernel with a radius of 2 pixels. Masks were visually inspected and Otsu’s threshold was adjusted when required. Cells were quantified from the masked DAPI images. A cell was defined as a region with more than 20 and less than 100 connected pixels. Regions with less than 20 connected pixels were ignored. The number of cells in regions with more than 100 connected pixels was estimated by dividing the number of connected pixels by 100 - the maximal number of connected pixels defined per cell. DAPI cells expressing GFP were quantified from the corresponding GFP BW mask. A cell expressing GFP was defined as a DAPI cell region with connected GFP pixels. The percentage of c-*fos* expressing cells (a DAPI cell region with connected GFP pixels) was computed in 512 μm x 512 μm regions of interest in the cortex underneath the lateral electrode, the cortex between the electrodes, and the dentate gyrus region of the hippocampus, as well as the corresponding contralateral cortical and hippocampal regions.

##### Brain Slice Montages

GFP greyscale images were converted to RGB images. Images from a single brain slice were visually rearranged and overlapped to form a montage of the imaged slice.

#### In-Vivo Stimulation in Awake Mice Followed by Staining for Cell and Synapse Markers

##### Surgical Procedure And Animal Habituation

On the day of the surgery, the mice were injected with Meloxicam (1mg/kg) and buprenorphine (0.1mg/kg) and were anesthetized with 1%–2% (vol/vol) isoflurane in oxygen and placed in a stereotactic frame. The scalp was shaved, ophthalmic ointment (Puralube Vet Ointment, Dechra) was applied to the eyes, and Betadine and 70% ethanol were used to sterilize the surgical area. The scalp was opened and a custom stainless steel headplate was affixed to the skull using dental acrylic (C&B Metabond, Parkell), and the mice were then recovered. Headfixed awake mice were then habituated to restraint for three consecutive days for 15, 30 and 45 min respectively. During this time the animals were placed in a cylinder-shaped tube. Animals were rewarded with sweetened condensed milk (diluted 1:2 in water) every 5-10 min during habituation.

##### In Vivo Transcranial Stimulation

On the day of stimulation, part of the dental cement was removed to enable cranial electrode placement. The stimulation procedure was as described in the ‘In-vivo stimulation followed by c-Fos staining’ section, but electrodes were made of conductive paste (Ten20, Weaver and Company) instead of polyimide tubes filled with saline. The center of one cranial electrode was placed at a point at the midline and −1.5 mm anteroposterior from bregma, and the center of the second cranial electrode was located laterally at +2 mm mediolateral from bregma and at the same anteroposterior coordinate. Each cranial electrode was paired with a larger (approximately 8 mm diameter) electrode that was made of the same conductive paste and was located on the ipsilateral cheek. The complex impedance between each pair of electrodes was established by applying short currents of low amplitude (10 Hz and 2 kHz, 10 μA, 0.5 s) and measuring the applied current and observed voltage waveforms. The stimulation protocol comprised 10 s intervals of electrical stimulation with 0.25 s ramp-on and ramp-off periods, with 10 s rest intervals in between, over a 20 min period. The lateral electrode was driven at a frequency of 2 kHz and current amplitude of 125 μA and the medial electrode was driven at a frequency of 2.01 kHz and a current amplitude of 125 μA. In the case of Sham stimulation, the amplitude of both currents was set to 0 μA; the rest of the procedure was identical.

##### Histology, immunohistochemistry, and imaging

Mice were sacrificed and perfused (cold 4% paraformaldehyde in 1 x PBS) 24 hr later to assess cellular and synaptic integrity by labeling for the neuronal marker NeuN (1:1000, Synaptic Systems, #266004), astrocyte marker GFAP (1:500, Cell Signaling Technology, #12389), microglia marker Iba1 (1:500, Wako Cehmicals, #019-19741), presynaptic marker synaptophysin (1:500, SVP-38, Sigma, #S5768), apoptosis marker cleaved caspase-3 (1:250, Cell Signaling Technology, #9664) and the DNA damage marker γH2AX (1:500. anti-phospho-histone H2A.X, Millipore, #05-636). For immunostaining, 40 μm sections were incubated with blocking buffer (5% normal donkey serum and 0.3% Triton X-100 in PBS) for 1 hr. Primary antibodies were diluted in blocking buffer and incubated with the sections overnight at 4°C. Primary antibodies were visualized using the appropriate secondary antibody conjugates (Alexa Fluor 488, Alexa Fluor 594 and Alexa Fluor 647, ThermoFisher Scientific). We used the CK-p25 transgenic mouse, an established mouse model of neurodegeneration, which exhibits neuronal atrophy, reduced synaptic density and pronounced DNA damage (Cruz et al., 2003, Dobbin et al., 2013, Kim et al., 2008), as a positive staining control for yH2AX antibody staining. The samples were then washed, stained with DAPI (Sigma, #10236276001) and mounted onto glass slides. Images were acquired on a Zeiss LSM710 laser-scanning confocal microscope using 20x and 40x air objectives, and subsequently analyzed in ImageJ.

#### In-Vivo Temperature Measurement

##### Surgical Procedure

Surgical procedures were as described in the ‘In-vivo stimulation followed by c-Fos staining’ section.

##### In Vivo Transcranial Stimulation

Stimulation currents ($I_{1}$, 2 kHz, 500 μA; $I_{2}$, 2 kHz, 500 μA) were simultaneously applied with 0.5 ramp-up and ramp-down periods via cranial electrodes that were configured on the skull as described in the ‘In-vivo stimulation in awake mice followed by staining for cell and synapse markers’ section.

##### Intracranial Temperature Measurement

A 1 mm diameter thermocouple (type “K” dual 36 Gauge with Teflon insulation. 36 TT-K-36, OMEGA Engineering) was inserted to the brain underneath the lateral electrode via a 2 mm diameter craniotomy, and continuous temperature measurements were obtained with a temperature logger (NI USB TC01, National Instruments) during 60 s of stimulation as described above, as well as during 30 s periods before and after stimulation.

#### In-Vivo Stimulation with Spatial Probing of Motor Cortex Functionality

##### Surgical Procedure and In Vivo Transcranial Stimulation

On the day of the experiment, mice were anesthetized with 100 mg/kg ketamine and 10 mg/kg xylazine. Ophthalmic ointment (Puralube Vet Ointment, Dechra) was applied to the eyes. The scalp was shaved and sterilized with Betadine and 70% ethanol and the mice were headfixed. Two electrically isolated currents $I_{1}$ and $I_{2}$ were applied transcranially via electrodes that were made of conductive paste (1 mm diameter; Ten20, Weaver and Company) and connected to the stimulator via thin silver wires. Current $I_{1}$ was applied via the skull electrode that was located at coordinates relative to bregma AP −1.5 mm, ML +2 mm (n = 5 mice) or ML −2 mm (n = 4 mice). Current $I_{2}$ was applied via the skull electrode that was located 2.5 mm laterally to the $I_{1}$ electrode (distance between centers of electrodes). Both skull electrodes were paired with a 5-8 mm diameter electrode, made of adhesive electrode gel (SignaGel, ParkerLabs), that was attached to the ipsilateral cheek. Stimulation blocks comprised a 0.5 s ramp-up period, a 6 s stimulation period and a 0.5 s ramp-down period. There was approximately 10 s interval between consecutive stimulation blocks.

##### Recording of Motor Activity

Motor activities were recorded using a video camera (Fujinon, YV10x5B-2, 1:1 3/5-50mm 1/3” CS), over a period lasting from 1.5 s pre-stimulation until 1.5 s post-stimulation. The period of stimulation was indicated to the camera with a green LED that was positioned behind a post to avoid a direct illumination of the animal eye.

##### Data Analysis

Motor activities were analyzed offline using MATLAB (MathWorks). Movements of the forepaws and whiskers were measured with the help of an in-frame ruler. In the case of whisker movement, we analyzed movements of the whisker that showed the maximal periodic movement amplitude during stimulation. Movements of the ears were scored by three independent researchers who were blind to the stimulation conditions, per the following scale: 0, no movement; 1, weak movement; 2, strong movement; 3, very strong movement. Prior to scoring movements, the researchers were shown one example video with a weak movement and one example video with a very strong movement, to help calibrate their numerical judgments (these training videos were not included in the analysis).

#### Phantom Electric Field Measurements

##### Phantom Construction

A phantom was constructed from a 50 mm diameter petri dish. We mounted sixteen 1 mm diameter silver wire electrodes with equal spacing along the circumference of the phantom (i.e., an interelectrode spacing of 9.8 mm). The electrodes were connected to a 24-channel adaptor box that was connected to the TI stimulator. The phantom was filled with sodium chloride solution. The salt concentration was adjusted until an inter-electrode impedance of 3 kOhm was measured between two opposite electrodes.

##### Electric Field Measurement

The electric field was measured using two orthogonal 3.6 mm-spaced dipole electrodes constructed from medical stainless steel needle electrodes. The location of the probe was adjusted across an 36 mm × 36 mm matrix with 6 mm steps using two large range motorized linear stages (Compumotor NEMA 23 types Model S57-51-MO, Parker Hannifin Corporation). The signal from each dipole electrode was fed into two separate custom built ultra-high input impedance differential electrometer type buffer amplifiers and then the outputs of these amplifiers were differentially fed into lock-in amplifiers (SR830, Stanford Research Systems) before readout with a digital multimeter (3457A, Hewlett Packard). The movements of the probe and the readouts of the measurements from the digital multimeter were controlled by a Labview script. The measurements at each location were averaged several times to reduce noise.

##### Post-processing

For 2D electric field maps, measurement points were linearly interpolated (with interpolation factor 2) using MATLAB’s interp2 function.

#### Electromagnetic FEM Simulation

##### Electromagnetic Field Computation

Electromagnetic simulations were performed on the Sim4Life platform (Zurich MedTech AG) using the ‘stationary current’ solver - a real valued quasi-electrostatic finite element method (FEM) solver for the ohmic current dominated regime. The simulation solved the equation ∇σ∇ϕ=0, where σ is the local electrical conductivity and ϕ is the electric potential from which the electric field and the current density can be obtained as $\vec{E}=-\nabla \phi$ and $\vec{j}=\sigma \cdot \vec{E}$ respectively. The solver is suitable for the frequencies used in this paper, as displacement currents can be neglected compared to ohmic ones. The solver discretizes the model using adaptive, rectilinear meshes. Tissue properties have been assigned according to the IT’IS Foundation database of tissue properties (Hasgall et al., 2015). The same conductivity values were used at all frequencies. The stimulating currents were normalized by integrating the normal current density over a surface surrounding an electrode. The simulations were performed with Dirichlet boundary conditions at active electrodes. The spatial distribution of the envelope modulation amplitude caused by temporal interference was computed from the fields of both electrode pairs using $\left|{\vec{E}}_{AM}\left(\vec{n,}\vec{r}\right)\right|=\left|\left|\left({\vec{E}}_{1}\left(\vec{r}\right)+{\vec{E}}_{2}\left(\vec{r}\right)\right)\cdot \vec{n}\right|-\left|\left({\vec{E}}_{1}\left(\vec{r}\right)-{\vec{E}}_{2}\left(\vec{r}\right)\right)\cdot \vec{n}\right|\right|$ where ${\vec{E}}_{1}\left(\vec{r}\right)$ and ${\vec{E}}_{2}\left(\vec{r}\right)$ are the fields generated by the first and second electrode pair, respectively, at the location $\vec{r}\left(x,y,z\right)$ and $\vec{n}$ is an unit vector along the direction of interest (e.g., normal to the surface). The maximal envelope modulation amplitude along any orientation which results from the vector fields ${\vec{E}}_{1}\left(\vec{r}\right)$ and ${\vec{E}}_{2}\left(\vec{r}\right)$ at location $\vec{r}\left(x,y,z\right)$ was computed. Assuming without loss of generality (as the numbering of the channels can be swapped and the sign of ${\vec{E}}_{2}$ can be inverted) that $\left|{\vec{E}}_{1}\right|>\left|{\vec{E}}_{2}\right|$ and that the angle α (angle between ${\vec{E}}_{1}$ and ${\vec{E}}_{a}$) is smaller than (π/2), the maximal modulation amplitude is obtained using:

$$\left|{\vec{E}}_{AM}^{max}\left(\vec{r}\right)\right|=\{\begin{array}{cc} \begin{array}{c} 2\left|{\vec{E}}_{2}\left(\vec{r}\right)\right|\\ 2\left|{\vec{E}}_{2}\left(\vec{r}\right)\times \left({\vec{E}}_{1}\left(\vec{r}\right)-{\vec{E}}_{2}\left(\vec{r}\right)\right)\right|/\left|{\vec{E}}_{1}\left(\vec{r}\right)-{\vec{E}}_{2}\left(\vec{r}\right)\right| \end{array} & \begin{array}{c} if\;\left|{\vec{E}}_{2}\left(\vec{r}\right)\right|<\left|{\vec{E}}_{1}\left(\vec{r}\right)\right|\text{cos}\left(\alpha \right)\;\\ otherwise \end{array} \end{array}$$

#### Phantom Model

The homogeneous phantom model consisted of a saline medium with a conductivity of σ=0.333S/m. The inhomogeneous (‘4-layer’) model consisted of scalp (d=0.05R, σ=0.333S/m), skull (d=0.085R, σ=0.0083S/m), cerebrospinal fluid (d=0.023R, σ=1.79S/m) and brain (d=0.83R, σ=0.333S/m) layers, where d is the layer thickness normalized to the overall sphere’s radius R.

##### Mouse Model

A computational mouse model (IT’IS Foundation, Male OF1 Mouse) was developed by segmentation of a male OF1 Mouse, weighing 35.5 g, according to the method described in (Kainz et al., 2006). The resolution of the model in the x, y, z directions was 42 μm, 42 μm and 700 μm, respectively. The model did not include CSF. The model was fitted with two small cranial electrodes with outer radius of 0.5 mm and inner radius of 0.17 mm filled with saline (σ=0.333S/m) and two large surface electrodes of radius 2.2 mm with conductive gel (σ=1.79S/m) on the ventral torso. The grid resolution in the electrode vicinity was high (0.04 mm) to properly resolve field gradients, while the coarsest grid step in the exposed area was in the order of 0.12 mm.

### Quantification and Statistical Analysis

The reasoning behind our sample sizes is not based upon a power analysis, since the goal was to create a new technology. As noted in (Dell et al., 2002), “In experiments based on the success or failure of a desired goal, the number of animals required is difficult to estimate...” As noted in the aforementioned paper, “The number of animals required is usually estimated by experience instead of by any formal statistical calculation, although the procedures will be terminated [when the goal is achieved].” These numbers reflect our past experience in developing neurotechnologies.

#### In-Vivo Rodent Electrophysiology

##### Definition of center and dispersion

Spike firing rate (Figure 1Ii), spike firing or bursting rate (Figure 1Iii): shown values are mean ± SD.

##### Definition of n

Number of cells.

##### Statistical test and definition of significance

Significance (p < 0.05) was characterized by one-way ANOVA followed by post hoc test with Bonferroni correction for multiple comparisons.

##### Randomization strategy

The order of stimulation conditions (i.e., 10 Hz stimulation, 2 kHz stimulation, and TI stimulation) was randomized.

##### Inclusion/exclusion of data

We included all cells that responded to a control 10 Hz stimulation apart from one neuron that was excluded from the hippocampus analysis due to an unphysiologically high rest potential of ∼−37 mV.

Statistical details can be found in the legends of Figure 1Ii and Figure 1Iii, the Results section TI Stimulation: Concept and Validation of Neural Firing Recruitment, and Table S1.

#### In-Vivo Stimulation Followed by c-*fos* Staining

##### Definition of Center and Dispersion

Percentage of c-*fos* expressing cells (a DAPI cell region with connected GFP pixels) was computed in 512 μm x 512 μm regions of interest: shown values are mean values ± SD.

##### Definition of n

Number of animals.

##### Statistical test and definition of significance

Significance (p < 0.05) was characterized by one-way ANOVA followed by post hoc test with Bonferroni correction for multiple comparisons.

##### Randomization Strategy

Each animal was exposed to a single stimulation condition (i.e., 10 Hz stimulation, 2 kHz stimulation, TI stimulation, or TI stimulation with a large inter-electrode distance).

##### Inclusion/Exclusion of Data

We included all animals that underwent the stimulation procedure.

Statistical details can be found in the legends of Figure 3 and Figure S4, the Results section Stimulation of Mouse Hippocampus but Not Overlying Cortex, and Table S2.

#### In-Vivo Stimulation in Awake Mice Followed by Staining for Cell and Synapse Markers

##### Definition of Center and Dispersion

Immunohistochemical: shown values are mean ± SEM.

##### Definition of n

Number of brain sections.

##### Statistical Test and Definition of Significance

Significance (p < 0.05) characterized by one-way ANOVA.

##### Randomization Strategy

Each animal was exposed to a single stimulation condition (i.e., TI stimulation or Sham).

##### Inclusion/Exclusion of Data

We included all animals that underwent the stimulation procedure.

Statistical details can be found in the legends of Figure 4, Figure S5, the Results section Safety Characterization of TI Stimulation, and in Table S3. In-vivo temperature measurement (related to Figure 4I).

##### Definition of Center and Dispersion

Instantaneous change in brain temperature from baseline: shown values are mean ± SD.

##### Definition of n

Number of animals.

##### Statistical test and definition of significance

Significance (p < 0.05) characterized by one-way ANOVA.

##### Randomization strategy

N/A.

##### Inclusion/exclusion of data

We included all animals that underwent the stimulation procedure.

Statistical details can be found in the legend of Figure 4I.

#### In-Vivo Stimulation with Spatial Probing of Motor Cortex Functionality

##### Definition of center and dispersion

Motor threshold (Figures 5B and 5C) shown values are mean ± SD; number of responsive animals (Figure 5 panels Di, Ei, and Fi); evoked movements (Figure 5 panels Dii, Eii, and Fii) shown values are mean ± SEM.

##### Definition of n

Number of animals.

##### Statistical test and definition of significance

Significance (p < 0.05) was characterized by one-way ANOVA followed by post hoc test with Bonferroni correction for multiple comparisons.

##### Randomization strategy

The order of stimulation conditions (i.e., TI stimulation at different difference frequencies, Figure 5B; TI stimulation at different carrier frequencies, Figure 5C; TI stimulation at different current ratios, Figure 5 panels Dii, Eii, and Fii) was randomized.

##### Inclusion/exclusion of data

We included all animals that underwent the stimulation procedure.

Statistical details can be found in the legend of Figure 5, the Results section Steerable Probing of Motor Functionality without Electrode Movement, and in Table S4.

All the statistical analyses in this manuscript were performed by using MATLAB statistics toolbox (Mathworks).

## Author Contributions

N.G. designed the TI stimulator device, simulations, and experiments and wrote the paper. D.B. designed and implemented the TI stimulator. N.G., S.B.K. and H.J.S. designed and conducted in-vivo rodent stimulation and electrophysiology experiments. N.G., S.B. K., and A.R. designed and conducted in-vivo rodent stimulation experiments followed by c-*fos* staining. N.G. and N.D. designed and conducted in-vivo rodent stimulation experiments with evoke motor activities. H.J.S. and N.D. designed and conducted awake in-vivo rodent stimulation experiments, and N.D. followed the experiments with staining and imaging for cell and synapses markers. A.M.C. and E.N designed, developed, and conducted electromagnetic FEM simulations in phantom and mouse models. N.K., L.H.T., and A.P.L. oversaw and designed experiments. E.S.B designed devices and experiments, wrote the paper, and oversaw experiments.

## Supplemental Information

Table S1. Interferential Neural Activation in Intact Mouse Brain, Related to Figure 1

Table S2. Summary of Group Mean c-fos Expression Fractions: % of DAPI-positive cells, Related to Figure 3

Table S3. Safety and Temperature Assessment, in Awake Mice, Related to Figure 4

Table S4. Interferentially Evoked Motor Activity, Related to Figure 5

Movie S1. Interferentially Evoked Motor Activity: Effect of Varying the Difference Frequency, Related to Figure 5

Movie S2. Interferentially Evoked Motor Activity: Effect of Varying the Carrier Frequency, Related to Figure 5

Movie S3. Interferentially Evoked Motor Activity: Effect of Varying the Current Ratio, Related to Figure 5
