# Supplementary material for: Noninvasive Deep Brain Stimulation via Temporally Interfering Electric Fields
Source: Cell. 2017 Jun 1;169(6):1029–1041.e16. doi: 10.1016/j.cell.2017.05.024 (PMC5520675; doi:10.1016/j.cell.2017.05.024)
Supplement: Table S1. Interferential Neural Activation in Intact Mouse Brain, Related to Figure 1 [file mmc1.pdf]

Cell, Volume *169*

Supplemental Information

Noninvasive Deep Brain Stimulation via Temporally Interfering Electric Fields

Nir Grossman, David Bono, Nina Dedic, Suhasa B. Kodandaramaiah, Andrii Rudenko, Ho-Jun Suk, Antonino M. Cassara, Esra Neufeld, Niels Kuster, Li-Huei Tsai, Alvaro Pascual-Leone, and Edward S. Boyden

SUPPLEMENTAL TABLES

Table S1. Interferential neural activation in intact mouse brain (Related to Figure 1)

Cortex

Group number	Group name	Group mean (± st.d.) firing frequency (Hz)
1	Firing rate during 10 Hz stimulation	9.97 (± 0.85)
2	Firing rate during 1 kHz + 1.01 kHz TI stimulation	10.2 (± 0.83)
3	Firing rate during 2 kHz + 2.01 kHz TI stimulation	9.7 (± 0.85)
4	Firing rate during 1 kHz stimulation	0.2 (± 0.45)
5	Firing rate during 2 kHz stimulation	0.4 (± 0.31)
6	Spontaneous firing rate	0.35 (± 0.6)

ANOVA firing rates, cortex

Source	SS	df	MS	F	Prob>F
Groups	1122	5	224.5	502.6	2.8E-38
Error	20.1	45	0.44		
Total	1142	50			

Post-hoc multiple comparison test for neural firing rates, cortex

Group A	Group B	Mean (Group A – Group B)	95% CI (Group A – Group B)		p-value
1	2	-1.39	-0.23	0.91	1.00E+00
1	3	-0.78	0.32	1.43	1
1	4	8.56	9.77	10.98	7.79E-27
1	5	8.41	9.57	10.72	2.19E-27
1	6	8.72	9.63	10.54	6.57E-32
2	3	-0.58	0.56	1.71	1.00E+00
2	4	8.75	10.01	11.26	1.16E-26
2	5	8.61	9.8	11.0	3.71E-27
2	6	8.91	9.87	10.84	2.84E-31
3	4	8.23	9.44	10.66	3.25E-26
3	5	8.09	9.24	10.39	9.45E-27
3	6	8.4	9.31	10.22	2.92E-31
4	5	-1.45	-0.2	1.05	1.00E+00
4	6	-1.17	-0.13	0.90	1
5	6	-0.89	0.07	1.03	1.00E+00

Hippocampus

Group number	Group name	Group mean (± st.d.) firing frequency (Hz)
1	Firing rate during 10 Hz stimulation	9.74 (± 0.49)
2	Firing rate during 2 kHz + 2.01 kHz TI stimulation	10.22 (± 0.61)
3	Firing rate during 2 kHz stimulation	0.2 (± 0.47)
4	Spontaneous firing rate	0.45 (± 1.06)

ANOVA firing rates, hippocampus

Source	SS	df	MS	F	Prob>F
Groups	816	3	272.1	390.83	2.09E-26
Error	23.7	34	0.691		
Total	839.8	37			

Post-hoc multiple comparison test for neural firing rates, hippocampus

Group A	Group B	Mean (Group A – Group B)	95% CI (Group A – Group B)		p-value
1	2	-1.74	-0.48	0.77	1
1	3	8.12	9.54	10.95	8.05E-19
1	4	8.17	9.26	10.36	5.93E-22
2	3	8.69	10.02	11.36	2.55E-20
2	4	8.76	9.75	10.73	3.74E-24
3	4	-1.44	-0.27	0.9	1
